# Impact of *Skills for Change Program* on metabolic control, diet and physical activity levels in adults with type 2 diabetes: A cluster randomized trial

**DOI:** 10.1371/journal.pone.0304639

**Published:** 2024-05-31

**Authors:** Habiba I. Ali, Latifa Baynouna Al Ketbi, Carine Platat, Hanan Abdl El Baki, Fadima Elmi, Wissam Ibrahim, Taoufik Zoubeidi, Ayesha S. Al Dhaheri, Leila Cheikh Ismail, Maryam N. M. Tariq, Usama Souka, Javed Yasin, Lily Stojanovska

**Affiliations:** 1 Department of Nutrition and Health, College of Medicine and Health Sciences, United Arab Emirates University, Al Ain, United Arab Emirates; 2 Ambulatory Health Care Services, Abu Dhabi Healthcare Services, Al, Ain, United Arab Emirates; 3 Department of Medicine, Lincoln Medical Center, New York, NY, United States of America; 4 Department of Analytics in the Digital Era, College of Business and Economics, United Arab Emirates University, Al Ain, United Arab Emirates; 5 Department of Clinical Nutrition and Dietetics, College of Health Sciences, University of Sharjah, Sharjah, United Arab Emirates; 6 Nuffield Department of Women’s & Reproductive Health, University of Oxford, Oxford, United Kingdom; 7 Department of Internal Medicine, College of Medicine and Health Sciences, United Arab Emirates University, Al-Ain, United Arab Emirates; 8 Institute for Health and Sport, Victoria University, Melbourne, Australia; Pelita Harapan University Faculty of Medicine: Universitas Pelita Harapan Fakultas Kedokteran, INDONESIA

## Abstract

**Background:**

Type 2 diabetes mellitus is highly prevalent in the Arab Gulf countries. Despite this, limited culturally-adapted lifestyle intervention studies have been conducted in this region.

**Methods:**

In this culturally adapted 12-month cluster randomized trial, 382 patients with type 2 diabetes, aged 20–70 years were recruited from 6 public healthcare centers (3 interventions and 3 controls) in Al Ain, United Arab Emirates. The primary outcome of this study was a change in hemoglobin A1c (HbA1c). The secondary outcomes were Body Mass Index (BMI), low-density lipoprotein (LDL), high-density lipoprotein (HDL), triglycerides, total cholesterol, dietary intake, and physical activity levels. A diet and physical activity intervention, guided by the social cognitive theory, was delivered individually and in group format to the intervention group. The control group continued receiving only their usual diabetes management care. The data were collected at baseline and 1 year after participation.

**Results:**

The mean baseline HbA1c levels of the control and the intervention groups were 7.45 ± 0.11% and 7.81 ± 0.11%, respectively. At the end of the 12-month intervention, there was no significant difference in the changes of mean HbA1c between the intervention and the control groups. On the other hand, BMI and daily caloric intake were significantly decreased in the intervention compared to the control group by 1.18 kg/m2 (95% CI: -1.78 − -0.60) and 246 kcal (95% CI: -419.52 − -77.21), respectively, after controlling for age, gender, education, marital status, duration since diabetes diagnosis, diabetes treatment, treatment clinic, and baseline values. Sitting time during the week-end was significantly lower, difference 52.53 minutes (95% CI: 93.93 − -11.14).

**Conclusions:**

This community-based lifestyle intervention for patients with baseline HbA1c <8% did not result in a significant decrease of HbA1c but reduced caloric intake, body weight, and weekend inactivity after controlling for the covariates.

**Trial registration:**

This trial was registered on February 11, 2020 with Clinicaltrials.gov (NCT04264793).

## Introduction

Diabetes Mellitus (DM) is a chronic disease, affecting 537 million adults across the globe, and over 73 million people in Middle East and North Africa region (MENA) alone, as reported by International Diabetes Federation (IDF) [1]. The estimated prevalence of diabetes in the MENA region in 2030 is 95 million [1]. The United Arab Emirates (UAE), one of the 21 countries of the IDF MENA region, has undergone rapid infrastructure development and economic progress following the oil discovery [2].

This economic surge led to urbanization, sedentary lifestyles, and changes in dietary patterns characterized by energy-dense foods [3–5]. According to a national survey by the Ministry of Health and Prevention, 67.9% of the population is overweight and 27.8% obese [6]. In parallel to the high prevalence of overweight and obesity, there has been an increase in non-communicable diseases, especially diabetes and its complications [3, 5]. These trends are closely linked to the increased prevalence of diabetes in the UAE, with an age-adjusted comparative prevalence of diabetes of 16.4% in 2021 and a projected prevalence of 17.2% in 2030 [1]. An analysis of the UAE National Diabetes and Lifestyle Study (UAEDIAB) showed that nearly 40% of adult Emirati nationals are estimated to have either diabetes or prediabetes [2].

Diabetes requires complex daily self-management activities and decisions [7]. According to the American Diabetes Association Standards of Care in Diabetes—2023, diabetes self-management education and support (DSMES) is a critical element of care for all people with diabetes and those at risk for developing the disease [8]. DSMES is a complete blend of educational, clinical, behavioral, and psychosocial aspects of care required for daily self-management and lays a foundation to assist people with diabetes to take daily self-care with certainty and enhanced outcomes [8, 9]. DSMES has elements related to lifestyle changes, including diet and physical activity. Referral to registered dietitian nutritionist for customized medical nutrition therapy(MNT) along with DSMES has been proposed [10]. Effective self-management education has been shown to improve lifestyle behaviors, such as healthy eating habits and physical activity [11–14]. The evidence of MNT in improving diabetes outcomes is well-established [10, 15, 16].

Culturally adapted programs have shown great importance and improvement in diabetes care and management [17–19]. Several culturally tailored programs have been adopted in the MENA region [20–25]. These studies assessed participants’ HbA1c, lipid profile, and weight or BMI and were shown to improve diabetes management. In the UAE, a self-management intervention in the chronic diseases clinics implemented by Al Ain Ambulatory Health Services (AHS) showed improvements in glycemic control, blood lipid profiles, and blood pressure. However, lifestyle-related factors of cardiovascular disease, such as diet and physical activity levels did not improve, possibly due to the limited access to dietitians and other health education specialists to reinforce the physicians’ messages [26]. Saadi and colleagues (2007) found that among people diagnosed with diabetes, only 44% reported seeing a dietitian and 26% of them reported that they did not follow any diet to manage their diabetes [27]. Similarly, Al Kaabi and colleagues reported that less than half (46%) of patients with diabetes attending health centers in Al Ain had ever received nutrition counseling from a dietitian [28]. Furthermore, although a concerning lack of physical activity was reported in the UAE [4], such community interventions for people with diabetes are limited in this population.

Diabetes education interventions have already been implemented in many countries. However, only a limited number of community-based nutritional education interventions focusing on Emirati adults with type 2 diabetes have been conducted to date [29–31]. Moreover, the specific challenges involved in implementing such interventions among populations in the Gulf region and among the Arab populations are yet to be examined. The *Skills for Chang*e Diabetes Nutrition Education Program was a one-year, community-based, cluster-randomized prospective study for Emirati adults with type 2 diabetes attending AHS health centers in Al Ain. The study was conducted in 2011–2013 and was the first community-based nutrition intervention for Emirati adults with type 2 diabetes. The intervention was guided by the Social Cognitive Theory and focused on improving the diet and physical activity levels of Emirati adults with type 2 diabetes to decrease sedentary lifestyles, improve food choices and clinical outcomes, including glycemic control and blood lipids. We hypothesized that the intervention would improve glycemic control, body weight, diet, and physical activity levels in the intervention cluster compared to the control cluster.

The primary outcome of this intervention was a change in hemoglobin A1c (HbA1c). The secondary outcomes assessed at 12 months were Body Mass Index (BMI), low-density lipoprotein (LDL), high-density lipoprotein (HDL), triglycerides, total cholesterol, and dietary and physical activity levels.

## Methods

### Study design

**Fig 1:**
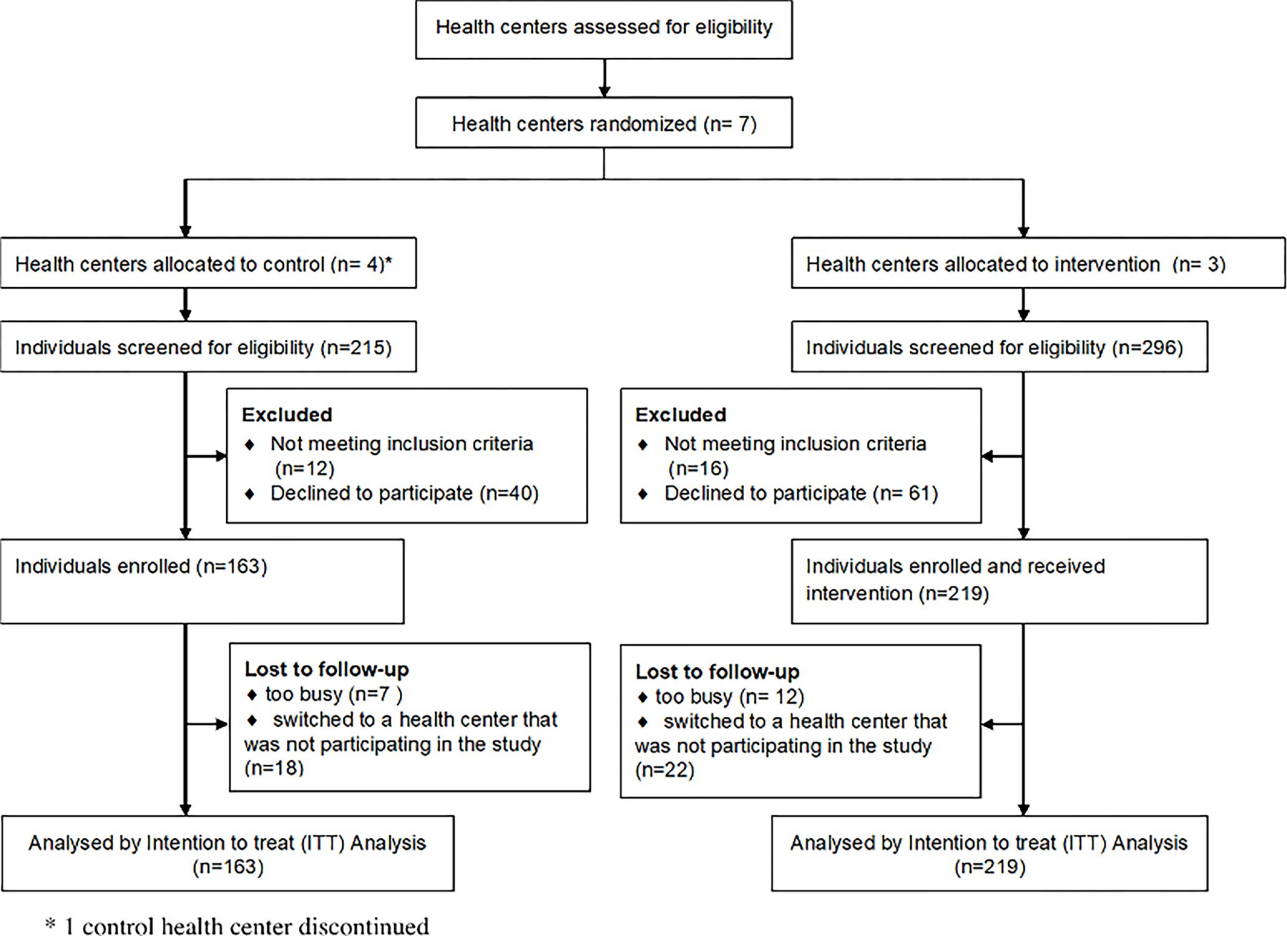
Consort statement showing participants enrollment, intervention allocation, follow-up, and data analysis throughout the study.

The *Skills for Change* Diabetes Nutrition Education Program was a cluster randomized trial where health centers in Al Ain, UAE, were the unit of randomization (clusters) (see S2 Table CONSORT statement for cluster randomized controlled trials). The seven health centers, managed by Al Ain Ambulatory Healthcare Services (AHS), were randomized to either an intervention or a control group. Cluster randomization was chosen to prevent contamination between the intervention and control participants within the same cluster (health center), and to ensure that health professionals did not provide intervention approaches to the control group attending the same health center. The assignment of the health centers to the intervention versus control groups was stratified by the center sizes and center locations. Of the 7 health centers, 3 were large urban centers, two were small urban centers, and two were large suburban centers. One large urban, one large suburban and two small urban centers were randomly assigned to the control group, and the remaining 3 centers were assigned to the intervention group (Fig 1).

Data collection followed a pre-post study approach where measurements were taken from participants at baseline and after one year from recruitment. The primary outcome of this intervention was a change in hemoglobin A1c (HbA1c). The secondary outcomes were Body Mass Index (BMI), low-density lipoprotein (LDL), high-density lipoprotein (HDL), triglycerides, total cholesterol, dietary intake, and physical activity levels.

The study aimed to achieve a change in one-year HbA1c values from baseline that is equal to one-third the standard deviation, which is approximately 1.8 based on data from patients with type 2 diabetes attending the AHS clinics in the Emirate of Abu Dhabi [32]. Thus, the absolute value of the decrease in HbA1c in the intervention group, 1 year after enrollment, is expected to exceed the corresponding value in the control group by 0.6. We chose to test this difference using the independent samples t-test with a power of 0.8 and a 2-tailed significance level of 0.05. If clustering effects were ignored, i.e., randomization was carried out on an individual basis, 144 participants per treatment group would be needed to achieve the desired power. On the other hand, when considering cluster effects and assuming a design effect of 2, the number of participants per group is doubled to approximately 290. However, we expected a gain in efficiency by adjusting for baseline values. Therefore, we anticipated that about 200 participants per group would be adequate (i.e., 200 for intervention and 200 for control).

### Setting

The study was carried out in AHS health centers in Al Ain, UAE. Al Ain is the second largest city in the Emirate of Abu Dhabi. According to the Statistics Centre-Abu Dhabi (SCAD), the total population of Al Ain in 2010 was 585,900 [33]. The target recruitment site of the participants was the 7 Primary Health centers under the management of AHS. These health centers provide comprehensive health care services ranging from walk-in acute conditions to regular follow-up of chronic diseases, such as diabetes and hypertension. From 2006, each of the health centers implemented a chronic disease clinic for patients with chronic non-communicable diseases, such as diabetes [26]. The chronic disease clinics located within each of these health centers were targeted to recruit potential participants for the study.

### Participants

The eligible study participants were all Emirati adults with type 2 diabetes, 20–70 years of age who were attending one of the 7 health centers in Al Ain, managed by the AHS. Exclusion criteria were participating in other nutrition intervention programs, very advanced age (>70 years), blindness, severe comorbidities, or any co-morbidities that may get worse by participation in the physical activity component of the intervention, such as heart failure.

Participant recruitment commenced on November 20, 2011 and ended in January 3, 2013. Physicians and nurses working in the chronic Disease Program clinics located in each of the health centers informed the patients about the program. Recruitment posters were also placed in the health centers. Verbal and written informed consent were obtained from all participants. The study protocol was approved by the Al Ain Medical District Human Research Ethics Committee (Protocol #:10/48). All procedures followed the principles of the Declaration of Helsinki. The trial was registered on February 11, 2020, with Clinicaltrials.gov (NCT04264793), https://clinicaltrials.gov/study/NCT04264793/. The research team did not anticipate the intervention presenting greater risks than those typically encountered in standard healthcare at these health centers. They adhered to all procedures specified in the approved research protocol by the Al Ain Medical District Human Research Ethics Committee despite retrospectively registering the intervention (see S2 File Skills for Change Protocol).

### Project advisory committee

A project advisory committee comprising academia and ambulatory healthcare services members from the three intervention health centers was formed to give guidance on various aspects of the program, including participant recruitment, data collection, and intervention implementation. The academic research team included a diabetes educator and physical activity expert. Representatives from the Ambulatory health care services included the management of the chronic disease clinics in the health centers, nurses, and dietitians working in the chronic disease clinics.

### Intervention

The intervention was modeled on social cognitive theory (SCT) [34] and employed several behavior modification techniques [35], including goal setting, self-monitoring, and building social support through group nutrition education and group physical activity sessions. It was delivered in participating health centers by health center chronic diseases nurses, dietitians, and research assistants from a local academic institution. All staff involved in the implementation of the intervention attended training workshops prior to the implementation of the intervention program. Physicians, nurses, and dietitians working in the three intervention health centers attended 6-hour workshop on diabetes nutrition counseling. Topics discussed in the workshops included: basic carbohydrate counting, adult education principles, and application of the relevant behavioral change theories in nutrition counseling including motivational interviewing [36, 37], stages of change model [38], and social cognitive theory [34]. Discussions, group activities, and role-pays involving case scenarios were used to practice the new skills. In addition, dietitians involved in dietary data collection attended 4-hour training on the use of the Multiple Pass Method for the 24-hour recall dietary interviews [39] to ensure data is collected in a standardized format.

### Components of the intervention

In addition to the routine medical care in the health center, the intervention included seven visits with the health center dietitian for individual nutrition counseling during the 12-month intervention. Participants were encouraged to follow diets emphasizing fruits, vegetables, and whole grains, and to decrease their intake of saturated fat and cholesterol. The dietary advice was individualized based on the 24-hour recalls obtained by the dietitian during the visit. Specific concepts of SCT [34] incorporated into the intervention to enhance program effectiveness included: self-monitoring, goal setting, and outcome expectancies [35]. The participants were encouraged to keep logs of blood glucose. During each visit with the dietitian, 1–2 behavior change goals addressing areas of concern identified during the nutrition assessment were discussed. Participants were given practical advice on achieving these goals. Progress towards achieving these goals was assessed during the following visits. In enhancing positive outcome expectations, health providers discussed the relationship between food choices, physical activity, and metabolic control. Culturally appropriate educational brochures on nutrition and physical activity were provided to the intervention participants. In addition, a video on health food shopping was produced and displayed in the waiting halls of the three intervention health centers. Moreover, participants were encouraged to attend at least three of the five group nutrition education sessions (S1 Table).

### Physical activity sessions

Individualized advice on ways of becoming more physically active was given by the AHS nurses working in the chronic disease clinics in the three intervention health centers. In addition, participants from these health centers were encouraged to join six-week physical activity group sessions. This program includes simple exercises (aerobic and strengthening) organized in three sessions with different durations and intensities that can be performed by any patient with type 2 diabetes irrespective of body weight. The first session was 15 minutes in duration and involved light-intensity exercises. The second was 30 minutes in duration and included light-to-moderate intensity exercises, and the third session was 60 minutes in duration and involved light-to-moderate intensity exercises. The group exercises were performed with the assistance of the Ambulatory Health Services nurses. Intervention participants were also provided with a DVD developed for Emirati patients with diabetes to encourage them to perform physical activity at home. To overcome the issue of a lack of group exercise facilities in two of the three intervention health centers, permission was obtained from local school authorities to conduct the group exercise sessions in schools near the health center. The participants in the control group received their usual care, including diabetes management education as part of the diabetes management from their physicians and nurses.

### Data collection

Trained staff in the health centers measured body weight (kg) and height (cm) of the participants dressed in light clothing and without shoes upon enrollment in the program. Body Mass Index (BMI) was calculated as weight (kg) divided by height squared (m^2^). Weight and height were measured using a SECA scale (Seca 799) with a stadiometer (Seca 220). Weight was measured to the nearest 0.1 kg and height to the nearest 0.5 cm. with participants wearing light indoor clothing and without shoes. Energy and nutrient intakes were assessed with a single 24-hour recall conducted at baseline and the end of the intervention. The 24-hour interviews were collected by trained dietitians using the Multiple Pass Method developed by the U.S. Department of Agriculture [39]. The multiple pass method uses multiple steps (or passes) in the interview process providing the respondent to remember food details and amounts. Participants were asked to recall all foods and beverages consumed during the 24 hours preceding the interview. The dietitians used food models, and household measures, such as cups and spoons to assist the participants in estimating food portion sizes consumed.

The International Physical Activity Questionnaire (IPAQ) Short Form [40] was used to measure physical activity levels. The IPAQ Short Form asks the respondent about the frequency (number of days and duration) during the past 7 days she/he has performed vigorous, moderate-intensity physical activities, or walking for at least 10 minutes. The participants were also asked about the time spent sitting during a weekday and a weekend day to assess physical inactivity (sedentarity).

The health center phlebotomist collected venous blood for biochemical measures (HbA1c, blood lipids). The samples were thoroughly mixed at room temperature and centrifuged immediately for 10 min at 4000 rotations/min. Plasma and serum were collected and measured using an automated analyzer Integra 400 Plus (Roche Diagnostics, Mannheim, Germany). All these measurements were collected at baseline and the end of the 1-year intervention.

### Dietary data analysis

Dietary data collected was analyzed in ESHA Processor, version 10.4. (ESHA Research) to calculate the energy and nutrient intakes of the participants. The ESHA food composition database was supplemented with food composition of composite dishes from regional food composition databases [37–39]. This was performed to ensure a culturally representative analysis of the 24-hour recall data. In addition, for the food items that were not included in these databases, recipe analyses were performed using the ESHA Food Processor (S1 File).

### Statistical analysis

Descriptive statistics were used to summarize the baseline characteristics of participants (means and standard deviations for continuous variables, and percentages for categorical variables). Comparisons of baseline continuous variables (age and diabetes duration) of the control and intervention groups were performed using an independent t-test, while comparisons between baseline categorical variables (sex, educational level, marital status, and diabetes treatment) were performed using the Chi-square test.

The effects of the intervention were analyzed using the intention‐to‐treat principle. Markov Chain Monte Carlo multiple imputations (MI) with predictive mean matching was used to handle missing data. The MI pooled mean and standard errors (SE) of the primary and secondary variables at baseline and after-one year were computed. Between-group comparisons of change in primary and secondary outcome variables were performed using independent samples t-test and mixed-effects multiple linear regression. When performing mixed-effects multiple linear regression the treatment clinic was considered a random effect (nested within treatments) while age, sex, education, intervention status, marital status, duration since diabetes diagnosis, diabetes treatment, and baseline value were considered fixed effects. Whenever the nested random effects were not significant, they were dropped from the model. The normality assumption of the mixed effect model was assessed using Shapiro-Wilk test. Residual plots were also used to check the other assumptions of the mixed-effects model such as homoscedasticity and linearity. The Box-Cox transformation was used to remedy for potential departures from the assumptions of the model.

All analyses were performed using the Statistical Package for Social Sciences (IBM SPSS Statistics for Windows, v. 28). P-values < 0.05 were considered statistically significant. Significance levels were not adjusted to account for the multiple comparisons, i.e., the multiple statistical tests, performed in this study.

## Results

Between November 2011 and January 2013, 382 individuals were recruited from 6 health centers, with (n = 3) for intervention and (n = 3) control centers (Fig 1). Due to a lack of recruitment, the 4^th^ control health center was discontinued. In total, 163 participants were recruited for the control group while 219 were recruited for the intervention group. Fifty-nine (15.4%) individuals were lost to follow-up (25 control and 34 intervention group). Data for 382 individuals was included in the intention-to-treat analysis (Fig 1). Missing values were estimated using multiple imputations.

The baseline characteristics of all participants are presented in Table 1. The mean age of the participants was 53.8± 9.47, with women accounting for more than half in both the control and intervention groups (72 and 78%, respectively). Most participants were married (85.1%) and did not receive any formal education (53.1%). The mean duration since the diagnosis of diabetes among all participants was 7.3 years with no significant difference between the control and intervention groups.

**Table 1:** Baseline characteristic of study participants (n = 382).

Variables	Total (n = 382)	Control (n = 219)	Intervention (n = 163)
Age^§^	53.84±9.47	54.92±9.96	53.04±9.02
Sex			
Female (%)	75.7	72	78
Male (%)	24.3		
Educational level			
No formal education (%)	53.1	49.1	56.2
Primary School (%)	20.4	23.9	17.8
Intermediate School (%)	10.7	16	6.8
High School or equivalent (%)	10.7	8.6	12.3
College/University (%)	5.0	2.5	6.8
Marital Status			
Single (%)	2.6	3.1	2.3
Married (%)	85.1	89.6	81.7
Divorced/Widowed (%)	7.4	7.4	16.0
Diabetes duration (years)^§^	7.51 ± 6.03	7.74 ±6.01	7.34 ± 6.06
Diabetes treatment			
No medication (%)	5.5	2.5	7.8
Oral medication (%)	86.4	90.2	83.6
Insulin only (%)	1.3	3.1	0.0
Insulin and oral medication (%)	6.8	4.3	8.7

^**§**^ Variables presented as mean ± SD.

Mean HbA1c of the control group decreased from 7.45% to 7.44% while mean HbA1c of the intervention group decreased from 7.81% to 7.64% (Table 2). However, there was no significant between-group difference in the changes of mean HbA1c (Adjusted difference: 0.10; 95% CI, −0.23 to 0.43; p = 0.545). The mean BMI of the control group increased from 31.84 ± 0.52 to 32.26 ± 0.57, while the mean BMI of the intervention group decreased from 32.09 ± 0.46 to 31.34 ± 0.45. The between-group difference in change of mean BMI was significant after controlling for age, sex, education, marital status, duration since diabetes diagnosis, diabetes treatment, treatment clinic, intervention status, and baseline BMI (-1.19; 95% CI, −1.78 to −0.60; P < 0.001). On the other hand, there were no significant between-group differences in changes of LDL, HDL, triglycerides, and total cholesterol before and after controlling for age, sex, education, marital status, duration since diabetes diagnosis, diabetes treatment, treatment clinic, intervention status, and baseline values.

**Table 2:** HbA1c, BMI and Lipid levels of study participants (n = 382).

Variables	Baseline^♢^	12 months^♢^[Table-fn t002fn001]	Change from baseline§	Between-group Adjusted Difference in change from baseline	P value ^¥^
				(95% CI)	
Primary Outcome: HbA1c (%)					
Control	7.45 ± 0.11	7.44 ± 0.13	-0.00 ± 0.12^**a**^	0.10	0.545
Intervention	7.81 ± 0.11	7.64 ± 0.11	-0.17 ± 0.14^**a**^	(-0.23−0.43)	
Weight (kg)					
Control	77.98 ± 0.51	78.86 ± 0.52	0.88 ± 0.25 ^**a**^	-2.74	0.011
Intervention	78.98 ± 0.34	77.66 ± 0.34	-1.87 ± 0.15^**b**^	(-4.86- -0.62)	
BMI (kg/m^2^)					
Control	31.84 ± 0.52	32.26 ± 0.57	0.42 ± 0.27^**a**^	-1.18	<0.001
Intervention	32.09 ± 0.46	31.34 ± 0.45	-0.75 ± 0.14^**b**^	(-1.78 − -0.60)	
LDL (mmol/L)					
Control	2.47 ± 0.08	2.53 ± 0.07	0.07 ± 0.10^**a**^	-0.01	0.912
Intervention	2.54 ± 0.06	2.58 ± 0.07	0.04 ± 0.09^**a**^	(-0.24 − 0.22)	
HDL (mmol/L)					
Control	1.09 ± 0.03	1.15 ± 0.02	0.06 ± 0.03^**a**^	-0.01	0.868
Intervention	1.06 ± 0.02	1.17 ± 0.02	0.10 ± 0.02^**a**^	(-0.11− 0.09)	
Triglycerides (mmol/L)					
Control	1.53 ± 0.18	1.46 ± 0.06	-0.07 ± 0.18^**a**^	0.18	0.272
Intervention	1.88 ± 0.19	1.61 ± 0.08	-0.27 ± 0.20^**a**^	(-0.15 − 0.51)	
Total Cholesterol (mmol/L)					
Control	4.38 ± 0.09	4.29 ± 0.08	-0.09 ± 0.11^**a**^	-0.01	0.954
Intervention	4.59 ± 0.12	4.38 ± 0.08	-0.21 ± 0.13^**a**^	(-0.34 − 0.32)	

^♢^ Pooled means and standard errors generated after multiple imputation of missing values.

^**§**^ Comparison performed using independent samples t-test. Different superscripts indicate significant differences (p<0.05).

^¥^ Mixed model multiple linear regression after controlling for age, sex, education, intervention status, marital status, duration since diabetes diagnosis, diabetes treatment, treatment clinic and baseline value.

HbA1c, hemoglobin A1c; BMI, body mass index; LDL, low-density lipoprotein; HDL, high-density lipoprotein.

The mean total daily calorie intake of the control group increased from 1443.02 ± 48.83 to 1688.44 ± 51.45 kcal, while the mean total daily calorie intake of the intervention group decreased from 1581.12 ± 49.10 to 1437.09 ± 36.83 kcal (Table 3). Between-group difference in change of mean total calorie intake was significant (p<0.001), using an independent t-test. This difference remained significant after controlling for age, sex, education, marital status, duration since diabetes diagnosis, intervention status, diabetes treatment, treatment clinic, and baseline total calorie intake (-248.36; 95% CI, -419.52 to -77.21; P  =  0.005).

**Table 3:** Macronutrient and food groups intakes of study participants (n = 382).

Variables	Baseline	12 months	Change from baseline^§^	Between-group adjusted difference in change from baseline	p value^¥^
				(95% CI)	
Total Calorie Intake (kcal)					
Control	1443.02 ± 48.83	1688.44 ± 51.45	245.42 ± 63.19^**a**^	-248.36	0.005
Intervention	1581.12 ± 49.10	1437.09 ± 36.83	-144.03 ± 61.24^**b**^	(-419.52 − -77.21)	
Percent calories from fat					
Control	20.81 ± 0.66	22.23 ± 1.04	1.42 ± 1.25^**a**^	-0.83	0.557
Intervention	23.6 ± 0.81	21.49 ± 0.77	-2.11 ± 1.14^**b**^	(-3.65–2.00)	
Percent calories from Saturated fat					
Control	7.29 ± 0.29	7.77 ± 0.28	0.48 ± 0.41^**a**^	-0.23	0.538
Intervention	7.45 ± 0.24	7.64 ± 0.22	0.19 ± 0.34^**a**^	(-0.96 − 0.50)	
Percent calories from Carbohydrates					
Control	64.80 ± 0.91	64.40 ± 1.05	-0.40 ± 1.42^**a**^	13.06	0.844
Intervention	62.04 ± 0.89	63.41 ± 0.87	1.37 ± 1.24^**b**^	(-3.65− 2.99)	
Percent calories from Protein					
Control	15.85 ± 0.41	15.04 ± 0.38	-0.81 ± 0.56^**a**^	1.50	0.039
Intervention	15.7 ± 0.36	16.74 ± 0.39	1.04 ± 0.55^**b**^	(0.08 − 2.92)	
Cholesterol per 1000 kcal (mg)					
Control	117.88 ± 12.25	103.29 ± 10.9	-14.59 ± 15.97^**a**^	7.67	0.764
Intervention	130.25 ± 10.68	125.32 ± 9.99	-4.93 ± 15.13^**a**^	(-42.79 − 58.12)	
Dietary Fiber (grams)					
Control	20.09 ± 1.25	20.32 ± 0.96	0.23 ± 1.37^**a**^	0.64	0.779
Intervention	19.37 ± 0.7	21.19 ± 0.86	1.82 ± 1.05^**a**^	(-3.84 − 5.12)	
Milk servings per day (Cups)					
Control	1.05 ± 0.15	1.1 ± 0.13	0.05 ± 0.18^**a**^	0.12	0.445
Intervention	0.91 ± 0.11	1.2 ± 0.12	0.29 ± 0.17^**a**^	(-0.20 − 0.45)	
Fruit servings per day (Cups)					
Control	1.4 ± 0.14	1.19 ± 0.11	-0.21 ± 0.16^**a**^	0.16	0.181
Intervention	1.1 ± 0.09	1.29 ± 0.08	0.18 ± 0.12^**b**^	(-0.08 − 0.40)	
Vegetable servings per day (Cups)					
Control	1.58 ± 0.24	1.59 ± 0.22	0.01 ± 0.31^**a**^	0.79	0.058
Intervention	1.53 ± 0.12	2.25 ± 0.16	0.72 ± 0.23^**b**^	(-0.03 − 1.6)	

^♢^ Pooled means and standard errors generated after multiple imputation of missing values.

^**§**^ Comparison performed using independent samples t-test. Different superscripts indicate significant differences (p<0.05).

^¥^ Mixed model multiple linear regression after controlling for age, sex, education, intervention status, marital status, duration since diabetes diagnosis, diabetes treatment, treatment clinic and baseline value.

Significant between-group differences in change of percent calories from fat (p<0.05) and change of percent calories from protein (p<0.05) were observed using an independent samples t-test. After mixed effect multiple linear regression, the difference in the change of percent calories from protein remained significant (1.50; 95% CI, 0.08 to 2.92; P  =  0.039) however, the between-group difference in change of percent calories from fat was no longer significant.

The intervention group reported a greater increase in grams of dietary fiber consumed (1.82 ± 1.05 vs 0.23 ± 1.37) and cups of vegetables consumed (0.72 ± 0.23 vs 0.01 ± 0.31) per day compared to the control group. However, these differences were not significant when compared using the mixed effect multiple linear regression.

Changes in the micronutrient intake of the study participants are presented in Table 4. The reported sodium intake of the control group increased from 2038.46 ± 109.55 mg to 2625.11 ± 236.48 mg while the sodium intake of the intervention group decreased from 2623.7 ± 138.36 mg to 2423.56 ± 167.21 mg. Between-group differences in change of sodium were initially significant (p<0.05) based on the independent samples t-test, however, the differences were no longer significant (-270.05; 95% CI -928.19 to 388.09; p = 0.418) based on the mixed effect multiple linear regression. At the end of the study, between-group differences in potassium, calcium, and vitamin C intake per 1000 kcal were significantly higher among the intervention group after controlling for the covariates (p<0.001, p = 0.008 and p = 0.037, respectively).

**Table 4:** Micronutrient intakes of study participants (n = 382).

Variables	Baseline^♢^[Table-fn t004fn001]	12 months^♢^[Table-fn t004fn001]	Change from baseline^§^	Between-group adjusted difference in change from baseline	p value^¥^
				(95% CI)	
Sodium (mg)					
Control	2038.46 ± 109.55	2625.11 ± 236.48	586.65 ± 252.64^a^	-270.05	0.418
Intervention	2623.7 ± 138.36	2423.56 ± 167.21	-200.14 ± 212.04^b^	(-928.19 − 388.09)	
Potassium per 1000 kcal					
Control	1359.42 ± 53.49	1275.46 ± 72.19	-83.96 ± 85.15^a^	613.63	<0.001
Intervention	1210.6 ± 36.50	1861.4 ± 90.67	650.8 ± 99.47^b^	(348.62 − 878.65)	
Calcium per 1000 kcal					
Control	385.18 ± 18.30	385.96 ± 28.77	-8.59 ± 30.60^a^	81.80	0.008
Intervention	357.87 ± 13.25	460.95 ± 30.75	98.54 ± 28.33^b^	(21.66 − 141.94)	
Magnesium per 1000 kcal					
Control	144.31 ± 4.45	155.92 ± 6.43	11.61 ± 8.12^a^	15.54	0.126
Intervention	156.65 ± 4.17	169.88 ± 6.16	13.24 ± 7.72^a^	(-4.42 − 35.5)	
Iron per 1000 Kcal					
Control	7.25 ± 0.2	7.03 ± 0.23	-0.22 ± 0.29^a^	0.49	0.167
Intervention	7.48 ± 0.15	7.71 ± 0.26	0.24 ± 0.31^a^	(-0.2 − 1.18)	
Vitamin A RAE per 1000 kcal					
Control	197.4 ± 29.75	196.24 ± 43.01	-1.16 ± 57.75^a^	85.58	0.290
Intervention	205.05 ± 17.39	295.59 ± 39.56	90.54 ± 43.79^a^	(-74.49 − 245.66)	
Vitamin B_12_ per 1000 kcal					
Control	1.71 ± 0.21	1.62 ± 0.45	-0.08 ± 0.50^a^	0.17	0.661
Intervention	1.36 ± 0.11	1.97 ± 0.33	0.61 ± 0.35^a^	(-0.66 − 0.99)	
Folate DFE per 1000 kcal					
Control	167.36 ± 12.3	118.01 ± 9.55	-49.35 ± 15.4^a^	16.19	0.250
Intervention	157.09 ± 8.17	132.75 ± 8.27	-24.35 ± 11.78^a^	(-11.53 − 43.92)	
Vitamin C (mg) per 1000 kcal					
Control	59.1 ± 5.32	44.07 ± 3.45	-15.03 ± 6.18^a^	14.03	0.037
Intervention	54.47 ± 3.53	58.62 ± 4.8	4.14 ± 5.72^b^	(0.84 − 27.21)	
Vitamin D (mcg) per 1000 kcal					
Control	1.30 ± 0.17	1.47 ± 0.27	0.17 ± 0.30^a^	0.17	0.661
Intervention	1.09 ± 0.11	1.59 ± 0.17	0.51 ± 0.22^a^	(-0.66 − 0.99)	
Vitamin E (mg) per 1000 kcal					
Control	1.20 ± 0.13	1.53 ± 0.17	0.32 ± 0.17^a^	0.18	0.525
Intervention	2.05 ± 0.17	1.65 ± 0.12	-0.40 ± 0.20^b^	(-0.39 − 0.74)	

^♢^ Pooled means and standard errors generated after multiple imputation of missing values.

^§^ Comparison performed using independent samples t-test. Different superscripts indicate significant differences (p<0.05).

^¥^ mixed model multiple linear regression after controlling for age, sex, education, intervention status, marital status, duration since diabetes diagnosis, diabetes treatment, treatment clinic and baseline value.

Physical activity levels and sitting times of the study participants are presented in Table 5. The intervention group reported a mean increase of 99.47 ± 48.54 min per week in vigorous and moderate activity time, 63.35 ± 17.53 min per week in walking time, and 162.82 ± 52.68 min per week in total activity time. On the other hand, the control group reported a decrease of these types of activities. Change of vigorous and moderate activity time, walking time, and total activity time were significant in between-group (p<0.05, p<0.001, and p<0.001, respectively), according to the independent samples t-test. However, these differences were no longer significant after controlling for age, sex, education, marital status, duration since diabetes diagnosis, diabetes treatment, treatment clinic, and baseline values.

**Table 5:** Physical activity levels and sitting times of study participants (n = 382).

Variables	Baseline^♢^[Table-fn t005fn001]	12 months^♢^[Table-fn t005fn001]	Change from baseline^§^	Between-group adjusted Difference in change from baseline	p value^¥^
				(95% CI)	
Vigorous and Moderate activity time (min per week)					
Control	146.1 ± 65.61	56.04 ± 19.65	-90.06 ± 66.29^a^	101.66	0.169
Intervention	87.21 ± 39.73	186.68 ± 26.59	99.47 ± 48.54^b^	(-43.08 − 246.39)	
Walking time (min per week)					
Control	290.67 ± 21.89	170.35 ± 17.54	-120.32 ± 28.26^a^	40.31	0.172
Intervention	137.42 ± 14.81	200.77 ± 14.12	63.35 ± 17.53^b^	(-17.7 − 98.33)	
Total activity time (min per week)					
Control	436.76 ± 72.35	226.39 ± 28.67	-210.38 ± 77.14^a^	123.54	0.199
Intervention	224.63 ± 45.78	387.45 ± 31.86	162.82 ± 52.68^b^	(-65.19 − 312.27)	
Weekday sitting time (min per day)					
Control	268.61 ± 16.84	291.24 ± 12.65	22.64 ± 20.33^a^	-23.76	0.420
Intervention	393.50 ± 18.23	292.55 ± 9.53	-100.96 ± 20.48^b^	(-83.62 − 36.1)	
Weekend sitting time (min per day)					
Control	264.59 ± 16.44	313.4 ± 14.64	48.81 ± 21.16^a^	-52.53	0.014
Intervention	507.68 ± 19.72	290.65 ± 11.06	-217.03 ± 21.61^b^	(-93.93 − -11.14)	

^♢^ Pooled means and standard errors generated after multiple imputation of missing values.

^**§**^ Comparison performed using independent samples t-test. Different superscripts indicate significant differences (p<0.05).

^¥^ mixed model multiple linear regression after controlling for age, sex, education, intervention status, marital status, duration since diabetes diagnosis, diabetes treatment, treatment clinic and baseline value.

The control group reported a mean increase of 22.64 ± 20.33 minutes per day in weekday sitting time and 48.81 ± 21.16 minutes per day in a weekend sitting time, while the intervention group reported a mean reduction of 100.96 ± 20.48 minutes per day in weekday sitting time and 217.03 ± 21.61 minutes per day in a weekend sitting time. After controlling for socio-demographic characteristics, diabetes duration, intervention status, diabetes treatment, and baseline values the between-group difference in change of weekday sitting time was not significant (-23.76; 95% CI, -83.62 to 36.1) while the between-group difference in change of weekend sitting time remained significant (-52.53; 95% CI, -93.93 to -11.14).

## Discussion

The *Skills for Change* program was a one-year community-based randomized cluster study to increase physical activity levels, modify diet, and improve metabolic outcomes of adults with type 2 diabetes in the UAE. Despite the high prevalence of diabetes and its complications in the UAE, there was a lack of community-based diabetes nutrition education studies conducted in a representative population in the country when the *Skills for Change* program was conducted in 2011–2013. At baseline, the glycated hemoglobin of the participants in both groups was <8%. Although a greater reduction of HbA1c was observed in the intervention group, there was no significant difference in its reduction after 12-month participation between the intervention and control groups. On the other hand, caloric intake, body weight, and weekend sitting time remained significantly reduced in the intervention group after controlling for the covariates.

After the implementation of the *Skills for Change* program, three studies assessing the impact of behavioral lifestyle education were conducted in the UAE [29–31]. These studies reported significant reduction of HbA1c in the intervention group. However, baseline HbA1c levels of the intervention group in these studies were at least 8.5%, indicating a higher baseline HbA1c compared to the *Skills for Change* participants. Similarly, intervention studies conducted in other countries in the Arab Gulf region showed a significant reduction of HbA1c, when the baseline values were above 8% [41–43]. Participation in a 6-month intervention study involving audio-visual messages via mobile phone integrated with peer group support for newly diagnosed patients with diabetes in Saudi Arabia found a significant reduction of HbA1c in the intervention compared to the control group [41]. An intervention implemented by the existing healthcare staff, such as dietitians in Oman among patients with diabetes attending primary health centers in which the baseline HbA1c levels of the intervention and comparison group were 8.1 and 7.8%, respectively, did not find a significant between-group difference after 12-month intervention [42]. In a single-center, controlled study conducted in Kuwait, patients with HbA1c above 7% showed the greatest decrease in HbA1c, although DSME was associated with HbA1c of 1.7% after the 12-month intervention [43]. The results of these studies suggest that the efficacy of community-based interventions would depend on the initial metabolic status, with a greater impact being observed among patients with the most unfavorable status and a possible cutoff set at 8% for HbA1c.

The *Skills for Change* intervention led to a significant reduction in body weight with a mean difference in BMI between the intervention and control group of 1.18 kg/m^2^. This reduction in BMI is reflected in the reported caloric intake with a mean difference of 248 kcal/day between the intervention and control participants. Our results are in contrast to a 12-month intervention conducted in Kuwait in which there were no significant changes in body weight or BMI among the intervention group [43]. A previous 6-month randomized lifestyle intervention in the UAE did not find a significant change in BMI or body weight [29]. Similarly, in a study conducted in Oman, there was no significant change in BMI or weight between the intervention and the control groups after 12-month intervention [42]. Moreover, a previous intervention among patients attending the same Ambulatory Healthcare Services centers that resulted in reductions in glycemic control, blood lipid profiles, and blood pressure, found no significant improvements in body weight or dietary practices [26]. The involvement of trained staff and the quality of nutrition counseling components of the *Skills of Change* program could have contributed to the significant weight loss observed among the intervention group. In addition to the lower caloric intake, participants in the intervention group reported significantly higher intake of potassium, calcium, and vitamin C per 1000 kcal, even after controlling for the covariates, indicating more nutrient-dense food intake compared to the control group.

In the present study, weekend sitting time was significantly decreased in the intervention participants compared to the control group and remained significant after controlling for the covariates. Providing education and practical physical activity sessions during the week as well as a DVD to practice at home may have encouraged the participants to be less sedentary during the weekend. This finding is consistent with a previous study conducted in Qatar [44]. In a cluster randomized controlled trial conducted in Oman, sitting hours/day among the intervention group was significantly decreased compared to the control group [42]. Since the amount of moderate to vigorous physical activity and walking in the present study did not significantly change, the participants may have replaced the sitting time with other low intense activities, such as cooking which are not captured by the IPAQ.

The above results indicate a lack of evidence supporting a statistically significant reduction in the primary outcome, HbA1c. On the other hand, participation in the *Skills for Change* program led to significant improvements in secondary outcomes, including body weight, dietary intake, and physical activity levels among the intervention group, thus supporting the hypothesis for the other study outcomes tested.

The present study showed no significant difference between the intervention and the control groups regarding sex or age distribution. The participants were primarily female in both the intervention and the control groups, consistent with previous research conducted in the same health centers, in which only 38% were men [32]. Despite a higher prevalence of diabetes in Emirati women than men (23% vs. 21%) [2], socio-cultural barriers hinder physical activity among Emirati women [4, 45, 46]. Moreover, the presence of comorbidities and diabetes-related complications may also negatively impact physical activity practices.

Regular follow-up of patients with diabetes in a self-management education has been associated with a greater improvement in diabetes metabolic control [47]. In the UAE, a retrospective study conducted at Dubai Diabetes Center found that patients who attended at least 4 follow-up visits in 12 months had significantly lower HbA1c levels compared to those with less regular follow-up visits [30]. An intensive diabetes education consisting of 5 days implemented by diabetes educators in Saudi Arabia after 1year resulted in a significant improvement in body weight, fasting blood sugar, blood pressure, and lipid profile, except HDL [25]. These findings highlight the importance of frequent DSME to observe a positive influence on disease outcomes in patients with diabetes. To facilitate regular participation in intervention programs, alternative ways of maintaining contact with patients who do not attend visits to the health facilities should be explored. A study by Farooqi and colleagues [48] reported a significant decrease in HbA1c from 10.3 ± 1.9% at baseline to 7.4 ± 1.5% after 3-month participation in telemonitoring devices of 38 previously lost to follow-up from a diabetes center as well as a weight loss 1.3 kg. Another intervention study involving diabetes education via WhatsApp to patients with diabetes in the UAE resulted in a significant improvement in glycemic control from baseline with no significant change in the control group (8.5% to 7.7% vs. 8.5 to 8.4%, respectively) after 6 months [31]. Smartphone use is now a common trend among most people and convenient to avoid barriers related to transportation to health centers for education. These findings indicate the potential improvements in metabolic control of people with diabetes in the UAE associated with the use of smart phones.

During the implementation of the *Skills for Change* intervention, several barriers related to recruitment, regular participation, and adherence to diabetes management advice were encountered. Since this was the first time an educational intervention of this nature was implemented in a community setting for Emirati patients with diabetes, it was particularly challenging to convince the participants of the need for more frequent follow-ups for educational purposes and re-assessments. Moreover, participants reported time constraints, transportation issues, unavailability of dietitians in the health centers, and difficulties in changing diet due to family preferences as previously reported [49]. The majority of the participants were women with childcare and other responsibilities at home. Most of them also depended on other family members for transportation, thus limiting their regular attendance to the educational sessions held in the health center. To overcome issues related to transportation, the participation in the physical activity sessions, walking groups were held in the neighborhood park of one of the health centers. In addition, a school near one of the intervention health centers was used for group physical activity sessions.

The important lessons gained from this study will be valuable in the implementation of diabetes nutrition education in the UAE and elsewhere in the Arab Gulf Region. For example, in the a culturally adapted physical activity sessions, appropriate for male and female patients, should be implemented at the usual healthcare center of the patients, in addition to developing tools that can facilitate exercising at home for patients with diabetes. Moreover, achieving greater improvements in metabolic control, physical activity, and dietary behaviors may require interventions of greater intensity and duration than the *Skills for Change* program. For example, implementing health coaching through a dedicated health educator team in health centers can lead to significant and enduring improvements in patients’ metabolic control and lifestyle behaviors. Since more than 80% of the participants had less than a high school education, educational materials, posters, and an educational video on healthy food shopping were developed and displayed in the waiting areas of the intervention health centers.

The main limitation of the *Skills for Change* program was that it was implemented only in one city of the UAE. In addition, the study did not include a maintenance phase to assess the long-term impact of the program on the outcomes after the study ended. Another potential limitation of the study is a recall bias related to the self-reported dietary intake. However, this was minimized by the use the multiple pass method developed by the U.S. Department of Agriculture [39] to standardize 24-hour dietary recall interviews. On the hand, our study has a number of strengths. It is one of the few studies conducted in the UAE and other countries in the Gulf region which showed a significant decrease in body weight of patients with diabetes. Moreover, to our knowledge, the “*Skills for Change*” is the only 12-month community-based diabetes nutrition education intervention in the UAE that targeted multiple health centers.

The *Skills for Change* program established an advisory committee consisting of representatives from academia and AHS to provide recommendations for the intervention’s development and implementation. The committee included a diabetes educator, an expert of physical activity, chronic disease clinic and health center managers, as well as nurses and dietitians working in the chronic disease clinics. This is considered as one of the strengths of the study. Another important strength of the *Skills for Change* program was the involvement of the healthcare professionals working in these health centers as well as their increased knowledge and skills related to patient counseling and nutrition education. This capacity-building approach has the potential for sustainable patient education involvement beyond the implementation period of the study. After the implementation of the *Skills for Change* program, the number of dietitians in the Ambulatory Health Care Services has been increased to facilitate timely and more efficient patient education. Finally, our study was the first community-based lifestyle intervention designed for Emirati adults with type 2 diabetes and provides important lessons for practice and future lifestyle interventions in the UAE.

Recommendations for future diabetes education interventions, include designing and implementing interventions with longer duration, adapted to different baseline metabolic status and cultural background, to assess the impact of diabetes education on the different aspects of diabetes self-management. These studies should also include a maintenance phase assessment to evaluate the long-term impact of the intervention. An interdisciplinary team consisting of health professionals and academic researchers has the potential to develop relevant, feasible, and evidence-based lifestyle interventions for persons with diabetes. Integrating physical activity sessions into the routine medical visit by offering patients an exercise session in addition to medical and dietitian consultations should be considered. Furthermore, using telemedicine messaging and use of the social media platforms, such as WhatsApp, can offer alternative ways of maintaining regular contact with persons with diabetes.

## Conclusion

This community-based 12-month lifestyle intervention for Emirati adults with type 2 diabetes with baseline HbA1c <8% did not improve significantly the glycemic control. However, there was a significant decrease in daily caloric intake, body weight, and weekend inactivity. Future diabetes education interventions should be tailored to participants with diverse metabolic statuses and cultural backgrounds and include a maintenance phase to assess the sustainability of intervention outcomes over time. Finally, although our analysis controlled for potential confounding effects of the observed variables, there might be some other confounders which were not collected.

## Supporting information

S1 Table Group education sessions. (PDF)

S2 Table CONSORT statement for cluster randomized controlled trials. (PDF)

S1 File Deidentified skills for change dataset. (CSV)

S2 File Skills for change protocol. (PDF)
