# Supplementary material for: Impact of Skills for Change Program on metabolic control, diet and physical activity levels in adults with type 2 diabetes: A cluster randomized trial
Source: PLoS One. 2024 May 31;19(5):e0304639. doi: 10.1371/journal.pone.0304639 (PMC11142497; doi:10.1371/journal.pone.0304639)
Supplement: S1 Table — (PDF) [file pone.0304639.s001.pdf]

Skills for Change **Program Group Nutrition Education Sessions**

Session	Topic	Discussion Points
**# 1**	Planning your meals	Interactive discussion on the relationship between healthy eating, metabolic control and health outcomes are highlighted. Concepts on healthy eating for diabetes, basic food groups, serving sizes, and healthful choices are discussed. Dietitian uses food models, pictures and samples of real food. The concept of the plate method for meal planning is introduced. Participants discuss how their typical food group distributions compare with the Plate Method.
	Why being active is beneficial for my health?	An interactive discussion will be organized with patients to identify the health benefits of regular practice of physical activity for people with type 2 diabetes
**# 2**	Managing your weight	Discussion focuses on food choices, food portions, emotional eating, healthful food choices and tips, and physical activity. Participants are invited to share what benefits they can expect from managing their weights
	What are the different types of physical activities I should do?	Description of the different types of physical activities and the different context in which physical activity can be practiced. The 3 types of activities included into the current guidelines for physical activity in adults - aerobic physical activity, resistance exercise and strength exercise- will be defined by using examples of activities and demonstration of these activities.<br><br>The concepts of frequency and intensity will be defined by using easy to understand explanations and words.<br><br>Recommendations on safe practice of physical activity (management of glycemia, shoes and feet…) for people with type 2 diabetes will be provided<br><br>The session will be an interactive discussion involving the patients on the type, frequency and intensity of activities the patient is used to do at this time
**# 3**	Estimating food portions	Dietitian discusses food portion estimation skills and demonstrates using real foods, food models, and household common measures (cups, spoons, etc.). Includes participant exercises involving estimations of

		different food portions, including UAE traditional food and composite dishes. Participants "guesstimate" before they actually measure and then compare answers.<br><br>Food models, samples of real foods, household measuring utensils (cups, spoon, etc.) are used.<br><br>Consistent carbohydrate intake is emphasized
	To be active in the daily life	An interactive discussion will be organized with the patients to identify some easy way to be active in the daily life and tips will be provided by the educator.<br><br>The role of family and friends as support will be emphasized
**# 4**	Choosing healthy fats	Importance of both type of fat and amounts for good health is discussed; Benefits of using herbs & spices instead of fat are emphasized. Participants share recipes and their ideas for modification. Low fat cooking demonstration is conducted (if facilities are available)
**# 5**	Understanding the food labels	Participants learn about the benefits of using food labels and practice how to read food labels or recognize food packages that are lower in fat; participants are invited for a supermarket tour as a follow practical session
	How to keep active in the long-term?	Patients will be encouraged to give some suggestions which could help them to maintain their level of physical activity and/or to increase it in the future.<br><br>Advices on the way of progressively increasing the amount of physical activity in terms of frequency, intensity and duration will described by the exercise educator
