# Supplementary material for: Impact of Skills for Change Program on metabolic control, diet and physical activity levels in adults with type 2 diabetes: A cluster randomized trial
Source: PLoS One. 2024 May 31;19(5):e0304639. doi: 10.1371/journal.pone.0304639 (PMC11142497; doi:10.1371/journal.pone.0304639)
Supplement: S2 Table — (PDF) [file pone.0304639.s002.pdf]

Table 1: CONSORT 2010 checklist of information to include when reporting a cluster randomised trial

Section/Topic	Item No	Standard Checklist item	Extension for cluster designs	Page No *
**Title and abstract**				
	1a	Identification as a randomised trial in the title	Identification as a cluster randomised trial in the title	1
	1b	Structured summary of trial design, methods, results, and conclusions (for specific guidance see CONSORT for abstracts)[1,2]	The cluster randomization design along with the number of clusters per treatment arm, study results and conclusions are described in the abstract.	1-2
**Introduction**				
**Background and objectives**	2a	Scientific background and explanation of rationale	Rationale and background of the study are described.	3-5
	2b	Specific objectives or hypotheses	The study objectives pertain to improvement of diet and physical activity levels, decrease of sedentary lifestyle, and improvement of clinical outcomes, including glycemic and blood lipid profiles at the individual level; the hypothesis to be tested is stated.	4-5
**Methods**				
**Trial design**	3a	Description of trial design (such as parallel, factorial) including allocation ratio	The clusters and the allocation of clusters to either intervention or control arm are described in the Study Design and Settings sections.	5-6
	3b	Important changes to methods after trial commencement (such as eligibility criteria), with reasons	Due to a lack of recruitment, the 4th control health center was discontinued. This information is mentioned in the first paragraph of the Results section. It is also indicated in Figure 1.	11
**Participants**	4a	Eligibility criteria for participants	Eligibility criteria for selecting participants within a cluster are described in the Participants section.	6-7

	4b	Settings and locations where the data were collected	See the Section Setting.	6
**Interventions**	5	The interventions for each group with sufficient details to allow replication, including how and when they were actually administered	A detailed description of the intervention and data collected are presented in the sections Intervention, Components of the Intervention, Physical Activity, and Data Collection.	7-10
**Outcomes**	6a	Completely defined pre-specified primary and secondary outcome measures, including how and when they were assessed	The primary and secondary outcomes are measured at the individual level. They are described in Study Design section.	5
	6b	Any changes to trial outcomes after the trial commenced, with reasons	No changes were made.	N/A
**Sample size**	7a	How sample size was determined	The number of study participants in each treatment arm was determined as discussed in the Study Design section and was based on expected changes of values in the primary outcome taking into consideration the cluster randomization design.	5-6
	7b	When applicable, explanation of any interim analyses and stopping guidelines	Interim analyses and stopping times were not considered in the design and trial implementation.	N/A
**Randomisation:**				
**Sequence generation**	8a	Method used to generate the random allocation sequence	The method to select the clusters assigned to each treatment arm is described in the Study Design section.	5-6
	8b	Type of randomisation; details of any restriction (such as blocking and block size)	Assignment of clusters to treatment arms was based on the cluster sizes and the location of the cluster (urban vs suburban health center). This is described in the Study Design section.	5-6
**Allocation concealment mechanism**	9	Mechanism used to implement the random allocation sequence (such as sequentially numbered	Allocation of individuals to treatment arms was based on clusters. This is described in the Study Design section. The	5-6

		containers), describing any steps taken to conceal the sequence until interventions were assigned	allocation to the intervention group versus the control group was not blind, i.e., both the participants and the treating staff knew whether a cluster was assigned to the intervention or control arm, see the Section Study Design.	
**Implementation**	10	Who generated the random allocation sequence, who enrolled participants, and who assigned participants to interventions	Replace by 10a, 10b and 10c	
	10a		The selection of the clusters and their allocation to treatment arms were done by the researchers (Principal Investigator and Statistician) and is described in Study Design section.	5-6
	10b		Physicians and nurses working in the chronic disease program in each health center (cluster) recruited patients into the program. See the Participants section.	6-7
	10c		Since clusters (clinics) were pre-assigned to either control or intervention, the consent of participants was sought after randomisation. Verbal and written consent was obtained from all participants who agreed to participate in the program. See Section Participants.	6-7
**Blinding**	11a	If done, who was blinded after assignment to interventions (for example, participants, care providers, those assessing outcomes) and how	The sampling design did not involve blinding at any level.	N/A
	11b	If relevant, description of the similarity of interventions	N/A	N/A

**Statistical methods**	12a	Statistical methods used to compare groups for primary and secondary outcomes	Independent samples t-test and Mixed-effects multiple linear regression were used for Between-group comparisons of changes in primary and secondary outcome variables. The latter method takes into consideration the clustering effect on the observed values.	10-11
	12b	Methods for additional analyses, such as subgroup analyses and adjusted analyses	Descriptive analysis of the control and the intervention groups were performed and provided in Table 1.	12
**Results**				
**Participant flow (a diagram is strongly recommended)**	13a	For each group, the numbers of participants who were randomly assigned, received intended treatment, and were analysed for the primary outcome	A total of 163 participants were recruited for the control group and 219 were recruited for the intervention group see Results section. This information is presented in the first paragraph of the Results section and in Figure 1.	11-12
	13b	For each group, losses and exclusions after randomisation, together with reasons	Fifty-nine (15.4%) individuals were lost to follow-up (25 control and 34 intervention group), see Results section and Figure 1.	11
**Recruitment**	14a	Dates defining the periods of recruitment and follow-up	Participants were recruited between November 2011 and January 2013, see Results section.	11
	14b	Why the trial ended or was stopped	N/A	N/A
**Baseline data**	15	A table showing baseline demographic and clinical characteristics for each group	The baseline characteristics of all participants are presented in Table 1.	12
**Numbers analysed**	16	For each group, number of participants (denominator) included in each analysis and whether the analysis was by original assigned groups	The numbers of participants in each group are shown in Table 1.	12
**Outcomes and estimation**	17a	For each primary and secondary outcome, results for each group, and the	See tables 2 - 5.	13-20

		estimated effect size and its precision (such as 95% confidence interval)		
	17b	For binary outcomes, presentation of both absolute and relative effect sizes is recommended	N/A	N/A
**Ancillary analyses**	18	Results of any other analyses performed, including subgroup analyses and adjusted analyses, distinguishing pre-specified from exploratory	N/A	N/A
**Harms**	19	All important harms or unintended effects in each group (for specific guidance see CONSORT for harms[3])	N/A	N/A
**Discussion**				
**Limitations**	20	Trial limitations, addressing sources of potential bias, imprecision, and, if relevant, multiplicity of analyses	Limitations include sampling from one city only and the lack of a maintenance phase to assess the long-term impact of the program on the outcomes after the study ended are acknowledged in the Discussion section.	24
**Generalisability**	21	Generalisability (external validity, applicability) of the trial findings	Given the adopted experimental design, the findings of this study will be valuable in the implementation of diabetes nutrition education in the UAE and elsewhere in the Arab Gulf Region.	24
**Interpretation**	22	Interpretation consistent with results, balancing benefits and harms, and considering other relevant evidence	See Discussion section.	20-25
**Other information**				
**Registration**	23	Registration number and name of trial registry		2,7
**Protocol**	24	Where the full trial protocol can be accessed, if available		Attached

**Funding**	25	Sources of funding and other support (such as supply of drugs), role of funders	Included in the Financial Disclosure section in the submission system (as per PLOS One requirements)	

** Note: page numbers optional depending on journal requirements*

REFERENCES

1 Hopewell S, Clarke M, Moher D, Wager E, Middleton P, Altman DG, et al. CONSORT for reporting randomised trials in journal and conference abstracts. *Lancet* 2008, 371:281-283

2 Hopewell S, Clarke M, Moher D, Wager E, Middleton P, Altman DG at al (2008) CONSORT for reporting randomized controlled trials in journal and conference abstracts: explanation and elaboration. *PLoS Med* 5(1): e20

3 Ioannidis JP, Evans SJ, Gotzsche PC, O'Neill RT, Altman DG, Schulz K, Moher D. Better reporting of harms in randomized trials: an extension of the CONSORT statement. *Ann Intern Med* 2004; 141(10):781-788.
