# Supplementary material for: Impact of Skills for Change Program on metabolic control, diet and physical activity levels in adults with type 2 diabetes: A cluster randomized trial
Source: PLoS One. 2024 May 31;19(5):e0304639. doi: 10.1371/journal.pone.0304639 (PMC11142497; doi:10.1371/journal.pone.0304639)
Supplement: S2 File — (PDF) [file pone.0304639.s004.pdf]

Study Protocol

Study Title: Skills for Change: Development, Implementation and Evaluation of a Community-based Diabetes Nutrition Education Program for Emirati Adults with Type 2 Diabetes

Project Summary:

Project Summary:

The United Arab Emirates (UAE) with a prevalence of 19.5% of diabetes is considered by the International Diabetes Federation as the country with the second highest prevalence of diabetes in the world. Diabetes is a major risk factor for coronary artery disease, a leading cause of death in the United Arab Emirates. Other complications associated with type 2 diabetes are neuropathy and microvascular damage leading to blindness, and end-stage renal disease. Effective diabetes self-management requires that persons with diabetes acquire the necessary knowledge and skills to change their nutrition-related behaviors for optimal metabolic control. Evidence from randomized controlled studies, observational studies, and meta-analyses support the effectiveness of medical nutrition therapy for diabetes management. Despite the high prevalence of diabetes and its complications in the UAE, very few diabetes education intervention studies have been conducted in representative populations in the country.

We propose a one-year, community-based, cluster randomized study involving 400 Emirati adults with type 2 diabetes that will focus on diet and physical activity to decrease sedentary lifestyle and improve food choices and clinical outcomes, including glycemic control and blood lipids. The primary outcome of the intervention is a change in HbA1c. We expect this project driven by theoretical principles of behavior change and education will enhance the diabetes-related knowledge, skills, and behaviors of the participants. The results of this project will serve as an important guide in the development and implementation of culturally adapted future diabetes management nutrition intervention programs for Emirati adults with type 2 diabetes. The study will be conducted in collaboration with Al Ain Ambulatory Healthcare Services, Abu Dhabi Healthcare Services.

Study Investigators: Habiba I. Ali (Principal Investigator), Assistant Professor, College of Food and Agriculture, United Arab Emirates University, Al, Ain, United Arab Emirates, Latifa Baynouna Al Ketbi, Consultant, Family Medicine, Ambulatory Healthcare Services, Abu Dhabi Healthcare Services, Al Ain, Carine Platat, Assistant Professor, College of Food and Agriculture, United Arab Emirates University, Al, Ain, United Arab Emirates, Wissam Ibrahim, Associate Professor, College of Food and Agriculture, United Arab Emirates University, Al Ain, United Arab Emirates, Taoufik Zoubeidi, Professor, College of Business, United Arab Emirates University, Al Ain, United Arab Emirates

Rationale & Background Information

Diabetes mellitus and its complications are major causes of morbidity and mortality and contribute substantially to healthcare costs. The United Nations General Assembly passed a resolution in 2007 recognizing November 14th as World Diabetes Day and it encouraged all member states to develop national strategies and policies for the prevention, treatment, and care of people with diabetes. According to the International Diabetes Federation, the United Arab

Emirates (UAE) has the second highest prevalence of diabetes (19.5%) in the world (IDF, 2007). The prevalence of diabetes for only the Emirati adult population is nearly 25% (Malik, et al, 2005). Persons with diabetes have a 2 to 4-fold greater risk of cardiovascular disease than those without diabetes (Roglic et al 2005). According to the Ministry of Health coronary artery disease is a leading cause of death in the United Arab Emirates (Department of Preventive Medicine, 2006) and diabetes-related heart problems were the biggest killer in the UAE in 2008, accounting for 31.6 per cent of deaths. Diabetes is also a major economic burden to the country ($100-$200 million annually) (GulfNews, Sept 14, 2009). A study involving a random sample of UAE citizens living in Al Ain found that only 33.3% of subjects with diagnosed diabetes achieved metabolic control (HbA1c <7%) to reduce their risk of diabetes related complications (Saadi, et al, 2007).

A recent review highlighted the high prevalence of overweight, obesity, and diet-related non-communicable diseases in the UAE population (Ng et al, 2010). Results from a community-based survey on risk factors for cardiovascular disease conducted among 817 national residents of Al Ain city found that high waist circumference was significantly associated with hypertension and high BMI with diabetes (Baynouna et al, 2009). A previous chronic disease management intervention in the Al Ain Ambulatory Healthcare Services health centers showed improvements in glycemic control, blood lipid profiles, and blood pressure. However, lifestyle-related factors of cardiovascular disease, such as diet and physical activity did not improve possibly due to the limited access to dietitians and other health education specialists to reinforce the physician messages (Baynouna et al, 2010). Saadi and colleagues (2007) found that among people diagnosed with diabetes, only 44% reported seeing a dietitian and 26% of them reported that they did not follow any diet to manage their diabetes.

Evidence from randomized controlled studies, observational studies, and meta-analysis supports the effectiveness of medical nutrition therapy for diabetes (Pastors et al, 2002; Pastors et al, 2003). Diabetes self-management interventions, including those related to weight management, and physical activity have been shown to improve patterns of diabetes management and metabolic control among adults with type 2 diabetes (Norris et al, 2002). Besides, metabolic control achieved through medical nutrition therapy, physical activity, and medications (when needed) improves the quality of life and treats and prevents diabetes complications (ADA, 2007). The UK Prospective Diabetes Study showed that for every percentage decrease in glycosylated hemoglobin (HbA1c), there was a 37% reduction in the risk for microvascular complications and a 21% reduction in the risk for any **- endpoint** or death related to diabetes (Stratton, 2000). Studies on diabetes medical nutrition therapy (MNT) reported reductions in HbA1c, ranging from 0.25% to 2.9%, depending on the type and duration of diabetes. Individual sessions of MNT in these studies ranged from 1to 5 hours or were a series of 10 to 12 group sessions (2 hours each) (ADA Evidence Analysis Library, 2010).

Although diets high in fresh fruits, vegetables, and whole grains and lower in saturated fat have protective effects from cardiovascular disease, the leading cause of mortality for people with diabetes, many people with diabetes may not be getting the daily recommended number of fruits, vegetables, and whole grains. However, studies conducted with other populations without type 2 diabetes reported strong correlates between nutrition knowledge, attitudes, perceived beliefs, barriers, and intake of fruits and vegetables among adults (Steptoe et al, 2004; Watters et al, 2007).

Physical activity plays a major role in the prevention and control of insulin resistance, pre-diabetes, and type 2 diabetes (Coldberg, 2007). One prospective cohort showed that walking at least 2 hours per week was associated with a reduction in the incidence of premature death by 39% - 54% from any cause and 34% - 53% from cardiovascular disease among patients with diabetes (Gregg et al, 2003). The American Diabetes Association (ADA) recommends that adults with type 2 diabetes accumulate at least 150 minutes of moderate-intensity aerobic exercise and 3 sessions of resistance exercise per week (Sigal et al., 2006).
The Cochrane Database of Systematic Reviews has evaluated the effectiveness of culturally appropriate diabetes health education as an important outcome measure in type 2 diabetes and has concluded that culturally appropriate diabetes health education at least for the short term has a positive impact on glycemic control, knowledge of diabetes, and healthy lifestyles (Hawthorne et al, 2008). Furthermore, self-management education improves glycemic control at immediate follow-up, and increased contact time increases the effect (Norris et al, 2002). However, Further research is needed to assess the effectiveness of self-management interventions on sustained glycemic control, cardiovascular disease risk factors, and ultimately, microvascular and cardiovascular disease and quality of life (Norris et al, 2001).

To increase the likelihood of changing lifestyle behaviors, interventions, such as diabetes education programs often incorporate behavior change theories (Vallis et al, 2003; Parchman et al, 2003), including the Transtheoretical Model (Prochaska & DiClemente, 1982) and the Social Cognitive Theory (Bandura, 1986). The Transtheoretical Model of Behavior Change (TTM) (Prochaska & DiClemente, 1982) provides a framework for predicting a client's willingness to change lifestyle behaviors and is useful in facilitating persons with diabetes to modify and maintain new behaviors. The Social Cognitive Theory (SCT) is helpful in understanding and predicting both individual and group behavior and identifying methods by which behavior can be modified or changed (Bandura, 2001). It provides a framework for designing, implementing, and evaluating programs and is useful in predicting diabetes self-management behaviors, such as diet, self-monitoring of blood glucose, and physical activity (Glasgow, et al, 2002; Hays & Clark, 1999).

Despite the high prevalence of diabetes and its complications in the UAE, many people with diabetes do not receive advice on diabetes management from dietitians (Afandi, et al. 2006). A recent study found that although the majority of the persons with diabetes attending health centers have uncontrolled diabetes and excess body weight, less than half of them (46%) had ever received nutrition counseling from a dietitian (Al-Kaabi, et al, 2008). Additionally, there is limited research on nutrition knowledge related to diabetes management of persons with diabetes in the United Arab Emirates. A recent study involving a convenient sample of Emirati and Omani adults with diabetes reported low nutrition knowledge related to carbohydrate-containing foods (Ali et al, 2008). However, the main limitation of this study was that it involved a relatively small sample of volunteers. Similarly, in the UAE, although a worrying lack of physical activity was pointed out (Mabry et al, 2010), large community interventions including physical activity to improve the management of type 2 diabetes are still missing leading to a huge gap in terms of adapted approach, education and actions in the field of physical activity in this local population.

Therefore, further studies involving larger, more representative samples of Emirati adults with diabetes are needed to gain a greater understanding of diet and diabetes self-management

behaviors of individuals with diabetes in the UAE. We propose a collaborative project driven by theoretical models of behavior change and adult education principles with Al Ain Ambulatory Healthcare Services to evaluate the feasibility and effectiveness of a culturally appropriate, community-based diabetes nutrition education program for Emirati adults with type 2 diabetes. The project aims to improve nutrition knowledge, promote healthy diets (increased fruit and vegetable intake, reduced intake of saturated fat), and increase physical activity among Emirati adults with type 2 diabetes. The impact of the intervention procedures on metabolic control, anthropometric measurements, and cardiovascular risk lipid profiles will be investigated.

Study Aims and objectives

The overall aim of this research project is to design and implement a community–based diabetes nutrition education program to improve diet and physical activity behaviors as well as diabetes-related clinical outcomes among Emirati adults with type 2 diabetes attending Ambulatory Healthcare Services clinics in Al Ain City. The specific objectives are to:

1. Conduct a one-year intervention to evaluate the impact of an educational program on energy and nutrient intakes, nutrition knowledge, physical activity, and diabetes treatment satisfaction among Emirati adults with type 2 diabetes
2. Evaluate the impact of an educational program on diabetes-related clinical outcomes, including glycemic control, anthropometric measurements, and blood lipid profiles in Emirati adults with type 2 diabetes

Relevance of the proposed work to the UAE community:

To our knowledge, this project is the first community-based intervention focusing mainly on diabetes nutrition education in the UAE and thus it can serve as an important guide in the development and implementation of future diabetes management intervention programs in the country. The potential Benefits of the intervention include (1) Improved nutrition knowledge and skills for diabetes care; (2) Healthier food choices and increased physical activity levels; (3) Improved clinical outcomes (blood glucose, body weight, and blood lipids); (4) Culturally-appropriate exercise video to increase the physical activity levels of adults with diabetes and families; (5) Model community-based diabetes nutrition intervention program that has the potential to improve participant nutrition knowledge and skills; (6) Appropriate recommendations for larger scale programs in the UAE that have the potential to improve nutrition knowledge, skills, and clinical outcomes of adults with diabetes;(7) Increased scientific knowledge: Identification of the potential impacts of culturally-adapted diet intervention and regular practice of moderate physical activity on metabolic parameters.

Methodology

Design and Participants:

The seven health centers managed by Al Ain Ambulatory Healthcare Services will be the target setting for this study. The primary end-point for this intervention is HbA1c. We aim at a difference in one-year HbA1c value of one-third of the standard deviation which is approximately 1.8 based on recent data from patients with diabetes attending Ambulatory Health Services clinics in the Emirate of Abu Dhabi. Thus, we expect a 0.6 decrease in HbA1c in the

intervention group. We chose to test this difference with a power of 0.8 and a 2-tailed significance level of 0.05. If randomization had been carried out on an individual basis, we would need 16 X 9 = 144 participants per group. However, we have to take into account group randomization. Assuming a design effect of 2, we need approximately 300 per group. However, there will be a gain in efficiency by adjusting for baseline values. Therefore, 200 participants should be adequate per group (i.e. 200 for intervention and 200 for control). Based on this calculation, the seven health centers were randomly assigned as follows: 3 intervention clinics (Al Yahar, Al Muweiji, & Hilli) and 4 control clinics (Al Maqam, Al Zakher, Niyadat, & Mezyad). The health center setting (urban vs. suburban) and size (large vs. small) were considered during the randomization. The assignment of the health centers to the intervention versus control groups was stratified by the center sizes and center locations. Of the 7 health centers, 3 are large urban centers, two are small urban centers, and two are large suburban centers. One large urban, one large suburban and two small urban centers were randomly assigned to the control group, and the remaining 3 centers were assigned to the intervention group.

Exclusion criteria are: Very advanced age (>70 Y), patients with severe complications, such as renal or heart failure, blindness, or other morbidities that can prevent active participation in the program. Moreover, patients with contraindications to the required physical activity may be excluded from the structured physical activity program upon the recommendation of their attending physicians. Verbal and written informed consent will be obtained from all the participants. Ethics approval for the project will be obtained from the relevant research ethics committees (UAE University Research Affairs and Al Ain Medical District Human Research Ethics Committee).

Study Duration and Phases:
The overall study duration is 24 months. It consists of three major phases (Fig. 1).

Phase 1: Preparation Phase (8 months):
Design questionnaires: In this phase, project questionnaires will be finalized.

1. Diabetes Treatment Satisfaction Questionnaires (DTSQ): The questionnaire will be used to assess diabetes-related treatment satisfaction (pre and post-intervention). The questionnaire was designed by Prof. Clare Bradley, University of London, UK, and has been previously validated in Saudi Arabia.
2. 24-hour dietary recall: This will be used to assess energy and nutrient intakes. This tool was used in a national nutrition survey conducted in the UAE in 2009/2010.
3. Diabetes knowledge: This questionnaire covers nutrition knowledge related to food sources of carbohydrates, fiber, fat, saturated fat, and cholesterol. This questionnaire was developed by the research team from the related literature (Ali et al, 2008; Talbot et al 1997). The questionnaire will be tested for face and content reliability and modified accordingly before the intervention starts.
4. Physical activity questionnaire: The International Physical Activity Questionnaire (IPAQ) – Short Form (Booth, 2000)

Research ethics approval: During the Preparation Phase ethical approval for the project will be obtained from the relevant research ethics committees (UAE University Research Affairs Research Ethics Committee and Al Ain Medical District Human Research Ethics Committee)
Staff training: All project staff will attend training to ensure that research procedures will be followed. Dietitians, nurse educators, and physicians who will be involved in the patient education will attend a 3-day training and orientation program. Topics that will be discussed include: An update on nutritional management of diabetes, adult education principles, and application of the relevant behavioral change theories in nutrition counseling (including motivational interviewing, stages of change model, and social cognitive theory), and physical activity counseling. The training will be conducted in the format of a workshop and will use skill-building teaching methods, such as case studies and role plays.

Phase 2: Intervention Phase (12 months):
After obtaining verbal and informed consent and immediately after recruitment, all research participants will undergo detailed nutrition and other baseline assessments (dietary, anthropometric, biochemical, diabetes treatment satisfaction, and physical activity levels). In this phase, a 12-month diabetes nutrition education intervention will be implemented in three Ambulatory Healthcare Services clinics in Al Ain. The impact of the educational intervention will be evaluated at the end of the 12-month intervention by comparing data obtained from intervention and "usual care" (control) health centers.

Phase 3: Post-Implementation Phase (4 months): This phase involves data entry, data analyses, and preparation of the project's final report.

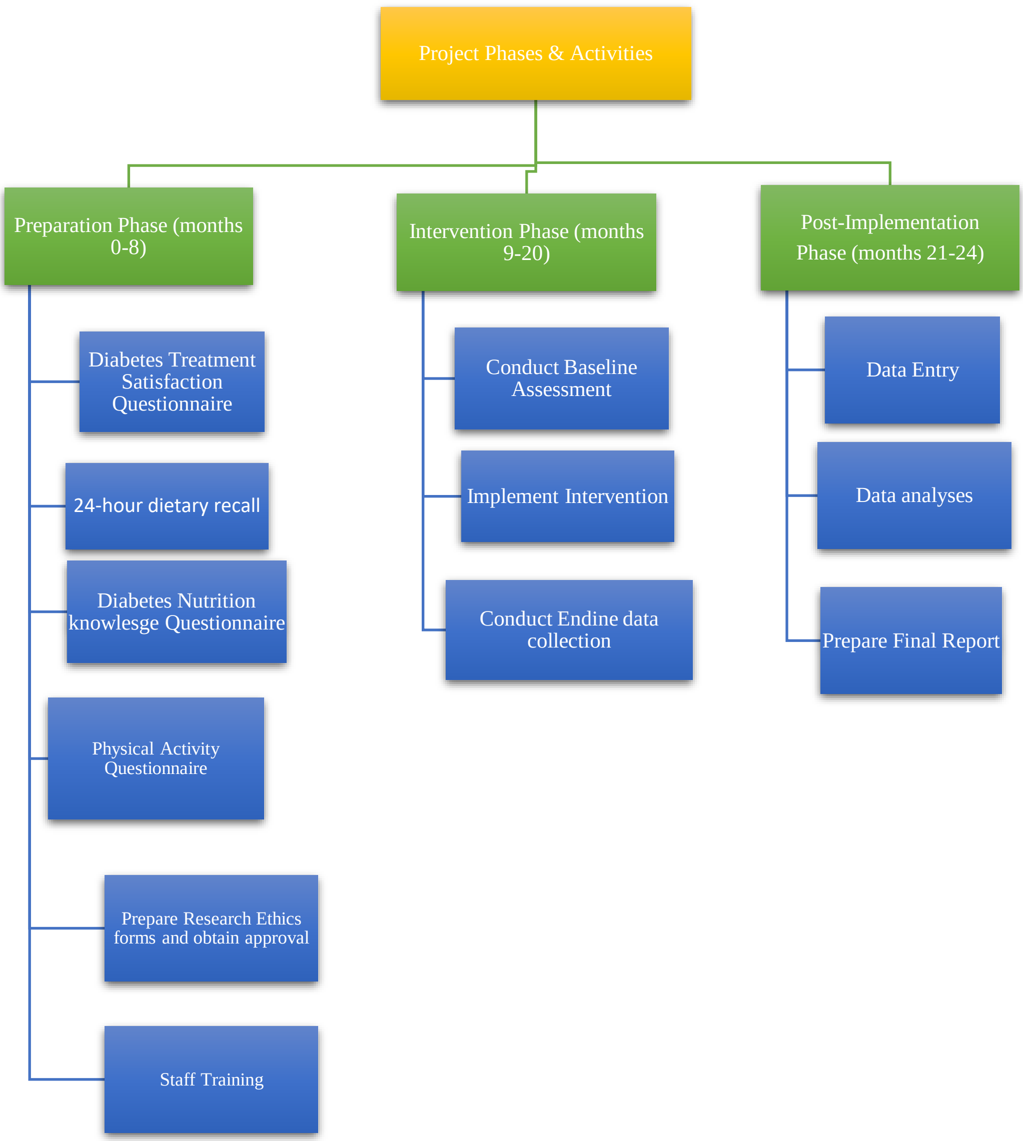

Fig. 1. Project Phases & Activities

Data collection:

The data collection plan at baseline and the end of the 12-month intervention is given in Table 1. The following data will be collected: (1) Energy and nutrient intakes, (2) Nutrition knowledge; (3) Physical activity levels; (4) Diabetes treatment satisfaction, (5) Anthropometric measurements (weight, height, Body Mass Index), (6) Biochemical assessments: HbA1c and blood lipids (total cholesterol, LDL-C; HDL-C, and triglycerides).

TABLE 1. Data Collection Schedule for Control (usual care) & Intervention Participants*

**Instrument/clinical parameter**	**Baseline (at recruitment)**	**Endline (after 12-month intervention)**
Anthropometric assessments: Height**, body weight	C, I	C, I
24-hour recalls	C, I	C, I
Diabetes Treatment Satisfaction Questionnaire (DTSQ)	C, I	C, I
International Physical Activity Questionnaire (IPAQ)	C, I	C, I
Biochemical parameters: HbA1c and blood lipids (LDL, TG, HDL)	C, I	C, I

* C – Control (usual care); I – Intervention subjects

**Height is measured only at baseline

Educational sessions for the intervention participants:

Research on diabetes self-management interventions has highlighted the important role of ongoing follow-up and support from professionals and members of the social network of the person with diabetes. For example, self-care education with telephone follow-up was associated with improved glycemic control (Piette et al, 2000).

Nutrition Education for the Intervention Group Participants:
Individual visits: Since contact time is an important predictor of outcome, participants in the intervention group will attend a total of 7 visits with the dietitian for individual nutrition counseling (20-30 minutes each). Nutrition counseling will be given monthly during the first 3 months and every 2 to 3 months for the remaining intervention period to improve glycemic control, dietary intake, and other outcomes. Participants will be encouraged to follow diets emphasizing fruits, vegetables, and whole grains and to decrease their intake of saturated fat and cholesterol. Dietary advice will be based on the 24-hour food recalls obtained by the dietitian during the visit. During each visit with the dietitian, behavior change goals addressing areas of concern will be identified and participants will be given practical advice in achieving the negotiated goals. Progress towards achieving these goals will be assessed during the following visits. Individualized educational strategies that employ adult education principles and behavior change theories, such as Social Cognitive Theory (Bandura, 1986), including, goal negotiation with the participant will be used to facilitate lifestyle behavior changes.

Group Education Sessions
Nutrition Sessions:
Intervention participants will be invited to attend a total of 5 group education sessions on diabetes management (diet & physical activity) conducted in the format of facilitated discussions that include interactive learning exercises. The group education aims to provide knowledge and skills as well as to build the social support network of the participants through regular contact with other people with diabetes and their healthcare team members. Topics for group education are given in Table 2 and include discussions on healthy eating for diabetes management, food portion estimation, food label reading activities, and other topics of relevance to the participants. Physical activity topics will to be covered, including the benefits of physical activity for people with diabetes and practical ways to enhance physical activity in daily life (climbing stairs instead of taking the elevator and performing stretching exercises while watching TV). The pleasure of practicing physical activity will be emphasized. Participants will be encouraged to attend at least three of the five group nutrition education sessions.

Table 2. Group Education Topics for the Intervention Participants

***Skills for Change* Program Group Nutrition Education Sessions**

Session	Topic	Discussion Points
**# 1**	Planning your meals	Interactive discussion on the relationship between healthy eating, metabolic control and health outcomes are highlighted. Concepts on healthy eating for diabetes, basic food groups, serving sizes, and healthful choices are discussed. Dietitian uses food models, pictures and samples of real food. The concept of the plate method for meal planning is introduced. Participants discuss how their typical food group distributions compare with the Plate Method.
	Why being active is beneficial for my health?	An interactive discussion will be organized with patients to identify the health benefits of regular practice of physical activity for people with type 2 diabetes
**# 2**	Managing your weight	Discussion focuses on food choices, food portions, emotional eating, healthful food choices and tips, and physical activity. Participants are invited to share what benefits they can expect from managing their weights
	What are the different types of physical activities I should do?	Description of the different types of physical activities and the different context in which physical activity can be practiced. The 3 types of activities included into the current guidelines for physical activity in adults - aerobic physical activity, resistance exercise and strength exercise- will be defined by using examples of activities and demonstration of these activities.

		The concepts of frequency and intensity will be defined by using easy to understand explanations and words.<br>Recommendations on safe practice of physical activity (management of glycemia, shoes and feet…) for people with type 2 diabetes will be provided<br>The session will be an interactive discussion involving the patients on the type, frequency and intensity of activities the patient is used to do at this time
**# 3**	Estimating food portions	Dietitian discusses food portion estimation skills and demonstrates using real foods, food models, and household common measures (cups, spoons, etc.). Includes participant exercises involving estimations of different food portions, including UAE traditional food and composite dishes. Participants "guesstimate" before they actually measure and then compare answers.<br>Food models, samples of real foods, household measuring utensils (cups, spoon, etc.) are used. Consistent carbohydrate intake is emphasized
	To be active in the daily life	An interactive discussion will be organized with the patients to identify some easy way to be active in the daily life and tips will be provided by the educator.<br>The role of family and friends as support will be emphasized
**# 4**	Choosing healthy fats	Importance of both type of fat and amounts for good health is discussed; Benefits of using herbs & spices instead of fat are emphasized. Participants share recipes and their ideas for modification. Low fat cooking demonstration is conducted (if facilities are available)
**# 5**	Understanding the food labels	Participants learn about the benefits of using food labels and practice how to read food labels or recognize food packages that are lower in fat; participants are invited for a supermarket tour as a follow practical session
	How to keep active in the long-term?	Patients will be encouraged to give some suggestions which could help them to maintain their level of physical activity and/or to increase it in the future.<br>Advices on the way of progressively increasing the amount of physical activity in terms of frequency, intensity and duration will be discussed

Physical Activity Sessions:
The participants will be invited to attend six-week exercise group sessions. The exercise classes, including basic resistance, flexibility, and aerobic exercises will be designed in collaboration with an exercise educator. Sessions will be offered separately for men and women by using indoor and/or outdoor exercise facilities. In addition; participants will be advised to perform exercise sessions at home (3 to 4 sessions per week) exercising at a level of 9 on the Perceived Exertion Scale. The amount of physical activity will be increased progressively through the intervention by encouraging the participants to perform longer and more intense sessions A video describing sessions, of different durations and at different intensities, providing some explanations and recommendations about the practice of physical activity will be given to each of the participants to help them practice of physical activity at home. This video will be produced in collaboration with UAEU Center for Educational Technology (CET). The physical activity program will be based on the theoretical principles of motivation, self-efficacy, goal-setting, and social support as well as on the simple premise that physical activity can be increased throughout the day at work, at home, and during leisure time. The educational component of physical activity will focus on both physical activity and sedentary behavior as described in the section above. Education will be combined with exercise training sessions. Group exercise sessions will be facilitated by a nurse and exercise specialist. The duration of each session will be 60-90 minutes.

Video on Healthy Food Shopping:
To enhance the practical application of nutrition counseling, a video on healthy food shopping will be produced in collaboration with Ambulatory Healthcare Services and will be displayed in the waiting halls of the three intervention health centers.

Facilitating nutrition-related behavior changes:
The intervention effectiveness will be enhanced by using individualized counseling, provision of sample meal plans and recipes, videos, and peer and professional support (frequent professional contact, group education). Specific concepts on Social Cognitive Theory (SCT) to be incorporated into the intervention to enhance program effectiveness include: (1) Goal setting (negotiate specific behavior change goals on diet and exercise); (2) Role modeling (providing guided practice on diabetes meal planning skills and opportunities for information sharing among participants during group sessions); (3) Enhancing social support peer support, and professional support); (4) Increasing self-efficacy (mastery learning through skill practice e.g. food label reading & food portion estimation skills); (5) Self-monitoring (encouraging participants to keep blood glucose logs); and (6) Enhancing positive outcome expectations (discuss the relationship between food choices, physical activity and metabolic control, relationship between food choices and health).

Intervention Monitoring and Evaluation:
A steering committee consisting of research team members from United Arab Emirates University and the Al Ain Ambulatory Health Services, Abu Dhabi Healthcare Services, will be set up to monitor the intervention implementation and evaluation. During the study, regular meetings will be held by the steering committee to discuss the various aspects of the study, including participant recruitment, intervention staff training needs, and ways of overcoming any potential difficulties faced during the study implementation.

Biochemical Methods

Blood samples will be drawn into dry and EDTA-containing tubes for obtaining serum and plasma respectively. Serum and plasma will be immediately separated after centrifuging the blood sample. Serum and plasma samples will be frozen at -80°C pending analysis. Serum samples will be used to measure the levels of HbA1c, triglycerides, total cholesterol, LDL cholesterol, and HDL cholesterol using a commercial system (UDICHEM ELITE; United Diagnostics Industry as described by Ibrahim et al. (2008).

Data analysis:

24-hour dietary recalls: The Food Processor software (ESHA Research, Oregon, USA) will be used to analyze the 24-hour diet recalls. Information from the existing regional and local food composition tables, such as the GCC (Gulf Cooperation Council) and Kuwait Food Composition Tables will be used to analyze the UAE traditional composite dishes. Recipe analysis by using the ESHA program will be conducted for UAE traditional composite dishes that contain different ingredients than the Kuwaiti composite dishes.

Statistical Analysis: Data will be analyzed using the Statistical Package for the Social Sciences, version 17.0 for Windows (SPSS, Chicago, U.S.A.). Data will be summarized by means, medians, standard deviations, interquartile range, and percentages as applicable. Differences between the intervention and control group related to the outcomes at 12 months will be tested, with a significance level of 0.05, considering the multilevel structure of the data.

Potential Benefits to Participants:

The study does not provide monetary or other compensation to the participants. However, there are other potential benefits as a result of participation in the intervention, including: (1) Improved nutrition knowledge and skills for diabetes self-management; (2) Healthier food choices; (3) Increased physical activity levels; (4) Improved clinical outcomes (blood glucose, body weight, and blood lipids); (5) Increased self-efficacy for adopting a healthy lifestyle; (6) Improved metabolic control may contribute to the prevention of chronic complications of diabetes.

Safety Considerations

There are no risks associated with participation in the study, except normal potential risks related to blood sample drawing which will be minimized by following standard protocols of the health centers. Blood collection will be performed by certified personnel assigned by the health centers to this task. Inconvenience related to the time spent on completing study questionnaires and attending educational activities will be minimized by arranging suitable times with the participants.

Informed Consent and Confidentiality:

Potential participants will be informed of the objectives and procedures of the study. They will be given the **Study Participant Information Sheet,** which includes: (1) The aim of the study; (2) the Data collection methods involved (blood collection and questionnaires); (3) the Right to refuse to answer any question (s) if desired; (4) Right to withdraw from the study at any time they want to. In addition, the participants from the intervention health centers will be informed about the intervention details, such as individual and group education involvement. All aspects of the study activities, including explaining the study purpose and other information related to the study will be conducted by research assistants fluent in Arabic. All potential participants will

be required to give informed consent (verbal and signed) for participation in the study. Strict confidentiality of the data collected will be maintained throughout the study duration. These procedures include the following:

- Names of the participants will not be recorded on the questionnaires or any of the forms; a unique ID# that matches the medical record ID# (chart #) will be used on all forms
- All completed forms/questionnaires will be kept in a locked filing cabinet
- When participants are to be called to remind their appointments, all calls will made be from the health center using their medical record contact information kept by the health centers; no other records of the contact information will be kept elsewhere.
- All staff involved in data collection will be required to observe strict confidentiality

Any problems arising during the implementation of the project will be reported to Al Ain Medical District Human Research Ethics Committee and the Ambulatory Healthcare Services.

Expected Outcomes of the Study

Relevance of the proposed work to the UAE community:
To our knowledge, this project is the first community-based intervention focusing mainly on diabetes nutrition education in the UAE and will serve as an important guide in the development and implementation of future diabetes management intervention programs in the country. Based on the lessons that will be learned from the study, nutrition education strategies found effective during the study will be used to incorporate into the educational program for people with diabetes in attending the health centers managed by the Ambulatory Healthcare Services.
Potential benefits of the intervention include: (1) Improved nutrition knowledge and skills for diabetes care; (2) Healthier food choices and increased physical activity levels; (3) Improved clinical outcomes (blood glucose, body weight, and blood lipids); (4) Culturally-appropriate exercise video to increase the physical activity levels of adults with diabetes; (5) Appropriate recommendations for larger scale programs in the UAE that have the potential to improve nutrition knowledge, skills, and clinical outcomes of adults with diabetes;(6) Increased scientific knowledge: Identification of the potential impacts of culturally-adapted diet intervention on metabolic parameters, physical activity, and dietary intake.

Dissemination of Results and Publication Policy

Annual reports will be submitted to the relevant human research ethics committees and required progress and final reports will be submitted to the study funding agency. The study results will be shared with Ambulatory Healthcare Services. The implementation process and the findings of the study will be shared with the scientific community through peer-reviewed publications and conferences.

Problems Anticipated

Potential challenges of the study implementation include recruitment and retention challenges of participants as well as adherence to the educational component of the study. Moreover, given this study will be the first nutrition intervention program for Emirati adults with type 2 diabetes, additional challenges may occur. Active involvement of the healthcare professionals working in these health centers in the study implementation as well as keeping frequent contact with the participants will be used to minimize these challenges.

Project Management

Contributions of the Research Team Members:

Dr. Habiba Ali, College of Food and Agriculture, United Arab Emirates University **(Principal investigator):** Dr. Ali will be responsible for the overall coordination of the project implementation. In addition, she will lead the development of the questionnaires and intervention nutrition education materials. She is a Registered Dietitian and Certified Diabetes Educator (Canadian Board). Dr. Habiba has expertise in the areas of nutrition education, diabetes education, and weight management.

Dr. Latifa Al Ketbi, Ambulatory Healthcare Services, Abu Dhabi Healthcare Services **(Co-investigator)**: Dr. Al Ketbi will be responsible for the project coordination and implementation in collaboration with United Arab Emirates University. She will play a key role in the study design and implementation. Moreover, she will liaise with the administrators of the Ambulatory Healthcare Services as needed.

Dr. Carine Platat, College of Food and Agriculture, United Arab Emirates University **(Co-investigator):** Dr. Platat will lead the physical activity component of the study. She will be responsible for the development of a specific physical activity program for patients with type 2 diabetes, staff training, and the measurements of the effectiveness of the physical activity program and its impact on patient health-related outcomes.

Dr. Wissam Ibrahim, College of Food and Agriculture, United Arab Emirates University **(Co-investigator):** Dr. Wissam will be responsible for evaluating the effects of the nutrition intervention on the biochemical parameters of the study.

Prof. Taoufik Zoubeidi, College of Business, United Arab Emirates University **(Co-investigator):** As a biostatistician, Dr. Zoubeidi will lead the statistical analysis and interpretation of the project data. In addition, he will guide the research design, sample size calculations, and advice to the study implementation.

Budget

The estimated budget needed for the study is itemized below:

	**Budget Category**	**in UAE Dirhams**
1.	Equipment	N/A
2.	**Consumables**	5,000
3.	**Chemicals & Drugs**	45,000
4.	Computer Costs	N/A
5.	**Research Assistants**	***111,750***
6.	Stationary	***500***
	**TOTAL**	***162,250***

Funding:

The financial support of the study will be covered by a grant from Sheikh Hamdan Bin Rashid Al Maktoum Award for Medical Sciences

References

ADA Evidence Analysis Library. Diabetes Mellitus (DM): Medical Nutrition Therapy. http://www.adaevidencelibrary.com/ Accessed November 8, 2010.

Afandi, B., Ahmed, S., Saadi, H., Elkhumaidi,S., et. al. Audit of a Diabetes Clinic at Tawam Hospital, United Arab Emirates, 2004–2005 Ann. N.Y. Acad. Sci. 2006;1084: 319–324.

Ali H, Bernsen RM, Taleb S, Al Azzan B. Carbohydrate – Food knowledge of Emirati and Omani Adults with Diabetes: Results of a pilot study. Int. J. Diabetes Metab. 2008;16: 23-26.

Al-Kaabi, J; Al-Maskar, F; Saadi, H; Afandi, B; Parkar, H; Nagelkerke, N. (2208) Assessment of Dietary Practice Among Diabetic Patients in the United Arab Emirates. Rev Diabetic Stud. 2008 Summer; 5(2): 110–115.

American Diabetes Association. Nutrition Recommendations and Interventions for Diabetes. Diabetes Care. 2007;Suppl.1; S48-65.

Bandura A. Social Foundation of Thought and Action. *1986.* Englewood Cliffs, NJ: Prentice Hall.

Bandura, A. (2001). Social cognitive theory: An agentive perspective. *Annual Review of Psychology, 52,* 1-26.

Baynouna LM et al A successful chronic care program in Al Ain-United Arab Emirates. BMC Health Serv Res. 2010 Feb 22;10:47.

Baynouna LM et al Associations of cardiovascular risk factors in Al Ain- United Arab Emirates., Cardiovasc Diabetol. 2009 Apr 16;8:21.

Berger G., Peerson A. Giving young Emirati women a voice: Participatory action research on physical activity. Health and Place, 2008;Epub ahead of print.

Booth, M. Assessment of physical activity: an international perspective. *Res. Q. Exerc. Sport* **71 Suppl 2**, 114–120 (2000).

Coldberg SR. Physical Activity, Insulin Action, and Diabetes Prevention and Control. Current Diabetes Reviews, 2007, 3, 176-184.

Department of Preventive Medicine, Annual Report 2004; Ministry of Health, Abu Dhabi, UAE, May 2006

Glasgow RE, Toobert DJ, Hampson SE, Strycker LA. Implementation, generalization and long-term results of the “choosing well” diabetes self-management intervention. *Patient Educ Couns.* 2002; 48:115-122.

Gregg E, Geerzoff R, Caspersen C, Williamson D, Narayan K. Relationship of walking to mortality among US adults with diabetes. *Arch Intern Med.* 2003; 163:1440-1447.

Hawthorne K, Robles Y, Cannings-John R, Edwards AGK. Culturally appropriate health education for type 2 diabetes mellitus in ethnic minority groups. *Cochrane Database of Systematic Reviews* 2008, Issue 3. Art. No.: CD006424. DOI: 10.1002/14651858.CD006424.pub2.

Hays LM, Clark DO. Correlates of physical activity in a sample of older adults with type 2 diabetes. Diabetes Care. 1999; 22:706-712.

International Diabetes Federation, Diabetes Atlas, third edition, 2007

Mabry RM, Reeves, MM, Eakin EG, Owen N. Evidence of physical activity participation among men and women in the countries of the Gulf Cooperation Council: a review. Obes Rev, 2010. 11(6):457-464.

Malik M, Bakir A, Abi Saab B and King H. Glucose intolerance and associated factors in the multi-ethnic population of the United Arab Emirates: Results of a national survey Diabetes Res Clin Pract. 2005; 69(2): 188-195

Ng SW, Zaghloul S, Ali HI, Harrison G, Popkin BM. The prevalence and trends of overweight, obesity and nutrition-related non-communicable diseases in the Gulf States. *Obes Rev.* Jun 10 2010; doi: 10.1111/j.1467-789X.2010.00750.x

Norris SL, Lau J, Smith SJ, Schmid CH, Engelgau MM. Self-management education for adults with type 2 diabetes: a meta-analysis of the effect on glycemic control. *Diabetes Care.* 2002;25:1159–1171.

Norris, SL, Engelgau MM, Narayan, KM. Effectiveness of self-management training in type 2 diabetes: a systematic review of randomized controlled trials. Diabetes Care. 2001 Mar;24(3):561-87.

Parchman ML, Arambula-Soloman TG, Noel PH, Larme AC, Pugh JA. Stage of change advancement for diabetes self-management behaviors and glucose control. *Diabetes Educ.* 2003;29:128-134.

Pastors JG, Franz MJ, Warshaw H, Daly A, Arnold MS. How effective is medical nutrition therapy in diabetes care? J Am Diet Assoc. 2003 Jul;103(7):827-31.

Pastors JG, Warshaw H, Daly A, Franz M, Kulkarni K. The evidence for the effectiveness of medical nutrition therapy in diabetes management. Diabetes Care. 2002 Mar;25(3):608-13.

Piette JD, Weinberger M, McPhee SJ, Mah CA, Kraemer FB, Crapo LM. Do automated calls with nurse follow-up improve self-care and glycemic control among vulnerable patients with diabetes? *Am J Med.* 2000;108:20–27.

Prochaska JO, DiClemente CC: Transtheoretical therapy: toward a more integrative model of change. *Psychother Theory, Res Pract* 1982;19:276-88.

Roglic G, Unwin N, Bennett PH, et al. The burden of mortality attributable to diabetes: realistic estimates for the year 2000. *Diabetes Care.* 2005;28:2130-2135.

Saadi, H., Carruthers SG; Nagelkerke, N., et al.   et al. Prevalence of diabetes mellitus and its complications in a population-based sample in Al Ain, United Arab Emirates. Diabetes Res. Clin. Pract.2007; 78; 369–377

Sigal R, Kenny G, Wasserman D, Castaneda-Sceppa C, White R. Physical activity/exercise and type 2 diabetes: A consensus statement from the American Diabetes Association. *Diabetes Care.* 2006;29:1433-1438

Steptoe A, Perkins-Porras L, Rink E, Hilton S, Cappuccio FP. Psychological and social predictors of changes in fruit and vegetable consumption over 12 months following behavioral and nutrition education counseling. Health Psychol. 2004;23:574-581.

Stratton IM, Adler AI, Neil HAW, et al. Association of glycaemia with macrovascular and microvascular complications of type 2 diabetes (UKPDS 35):Prospective observational study. Br Med J . 2000;321:405–12.

Talbot F, Nouwen A, Gingras J, Gosselin M, Audet J. The Assessment of Diabetes-Related Cognitive and Social Factors: The Multidimensional Diabetes Questionnaire. *Journal of Behavioral Medicine, 20 (3), 1997*.

Vallis M, Ruggiero L, Greene G, Jones H, Zinman B, Rossi S, Edwards L, Rossi JS, Prochaska JO. Stages of change for healthy eating in diabetes: Relation to demographic, eating-related, health care utilization, and psychosocial factors. D*iabetes Care.* 2003;26: 1468-1474.

Watters JL, Satia JA, Galanko JA. Associations of psychosocial factors with fruit and vegetable intake among African-Americans. *Public Health Nutr*. 2007;10:701-711.
